# Plasmon-Controlled Selective Emission Enhancement of Eu^3+^ with (Au Core)@(Y(V,P)O_4_:Eu) Nanostructures

**DOI:** 10.1021/acsnano.3c01462

**Published:** 2023-05-22

**Authors:** Jia Zhang, Xizhe Cheng, Han Zhang, Jiapeng Zheng, Jianfang Wang

**Affiliations:** † Physics Department and Jiangsu Key Laboratory of Modern Measurement Technology and Intelligence, Huaiyin Normal University, Huai’an 223300, China; ‡ Department of Physics, The Chinese University of Hong Kong, Shatin, Hong Kong SAR 999077, China; § School of Materials Science and Engineering, Zhejiang Sci-Tech University, Hangzhou 310018, China

**Keywords:** core@shell nanostructures, gold nanospheres, lanthanide ions, plasmon resonance, plasmon-enhanced photoluminescence

## Abstract

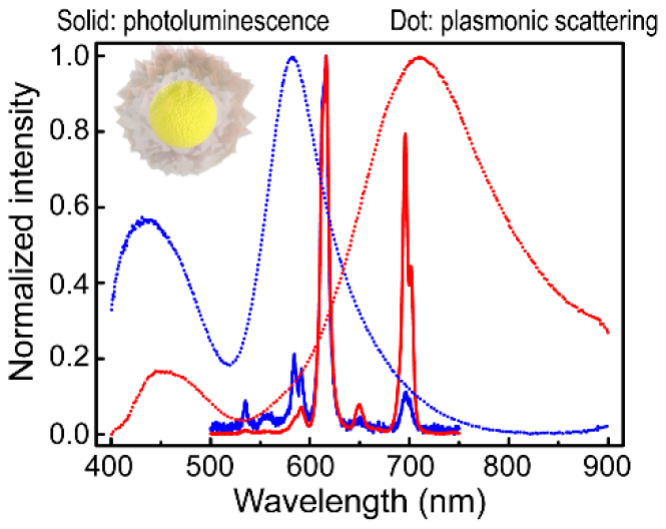

Plasmonic nanostructures have a capability to control the photoluminescence (PL) emission properties of optical species and thus can dramatically enhance the performances of diverse optical systems and devices. Lanthanide ions typically exhibit multiple PL emission lines. Systematic studies on the plasmon-enabled selective enhancement for the different emission lines of lanthanide ions are still highly desired in order to achieve the fine manipulation on the spectral profile and luminescence intensity ratio (LIR). Herein we report on the synthesis and PL emission properties of monodisperse spherical (Au core)@(Y(V,P)O_4_:Eu) nanostructures, which integrate the plasmonic and luminescent units into an individual core@shell structure. The localized surface plasmon resonance adjusted through control of the size of the Au nanosphere core enables the systematic modulation of the selective emission enhancement of Eu^3+^. As revealed by single-particle scattering and PL measurements, the five luminescence emission lines of Eu^3+^ originating from the ^5^D_0,1_ excitation states are affected by the localized plasmon resonance to different extents, which are dependent on both the dipole transition nature and the intrinsic quantum yield of the emission line. Based on the plasmon-enabled tunable LIR, high-level anticounterfeiting and optical temperature measurements for photothermal conversion are further demonstrated. Our architecture design and PL emission tuning results offer many possibilities for constructing multifunctional optical materials by integrating plasmonic and luminescent building blocks into hybrid nanostructures with different configurations.

Rare earth (RE)-doped luminescent nanoparticles belong to an attractive class of nanomaterials. They show great potential in diverse fields, including optical devices, biology, photodynamic therapy, temperature measurements, anticounterfeiting, lighting, and display.^1−5^ RE luminescence generally contains down-shifting, down- and up-conversion processes, which typically involve electric and magnetic dipole transitions. In many cases, trivalent RE ions suffer from low luminescence efficiencies due to the forbidden transitions among their 4f orbitals.^6^ To enhance and tailor RE luminescence for different applications, several strategies have been reported, including shell passivation, aliovalent doping, and use of metal-supported localized surface plasmon resonance (LSPR).^7−10^ As an important optical phenomenon, LSPR, usually brought about by noble metals, such as gold and silver, can dramatically alter the surrounding electromagnetic environment and interact with target objects such as RE ions, molecules, quantum dots, and fluorophores.^11−13^ In view of this, plasmon-enhanced photoluminescence (PL) has been widely investigated. Effective PL enhancement usually requires spectral overlap between the LSPR peaks of noble metals and the absorption/emission peaks of luminescent materials, known as excitation and emission enhancement, respectively.^14,15^ Many studies have been devoted to the plasmon-induced PL enhancement of RE upconversion processes in the past decades.^16−20^ The involved complicated energy transfer (ET) and cross-relaxation processes in the upconversion luminescence of RE ion pairs can make the understanding of the interactions between the electron populations and the plasmon resonance difficult and thus lead to difficulties in the control of spectral modification. On the contrary, the down-shifting luminescence of optical materials doped with a single type of RE ions undergoes a relatively simple electron transition process, which is similar to the case of well-studied fluorescent molecules. In this case, the control of the light absorption and emission rates becomes more expectable. Moreover, many important applications at present also involve down-shifting luminescence, such as lighting and display based on light-emitting diodes. The incorporation of plasmon resonance is very beneficial for improving the optical performances.

To yield high PL enhancement factors (EFs), the coupling of plasmonic nanoparticles and luminescent materials should be well designed. Several essential factors can affect the effective PL enhancement, including the LSPR peak shape and wavelength, the distance between the plasmonic nanoparticle and the emitter, and the plasmon resonance mode.^21−24^ Moreover, the LSPR wavelength of plasmonic nanoparticles strongly depends on the surrounding medium. For instance, a medium of a high refractive index usually leads to a red shift of the LSPR peak. To date, effective strategies have been developed to integrate plasmonic nanoparticles and RE-luminescent materials, such as the formation of hybrid core–shell-satellite nanostructures,^25^ template-assisted self-assembly into heterodimers,^26^ and heterointegration of luminescent nanocrystals with Au nanoshells.^27^ However, the previous studies have focused mostly on the effect of the metal nanoparticle shape and the distance with the emitter, as well as the emission enhancement for a particular peak or the full spectrum.^28−32^ Systematic studies on the selective PL enhancement of the different emission peaks from one RE activator have rarely been reported^33^ and are thus urgently needed. Such studies will deepen our understanding on the involved light–matter interaction and enable us to finely tailor the spectral profile and manipulate the luminescence intensity ratio (LIR). To this end, a single-particle spectral collection technique is highly favored. It offers several advantages. It can remove the averaging effect from different luminescent nanoparticles and the spectral information disorder that is caused by various incident directions of the excitation source against the particle surface. Importantly, such a technique enables direct precise inspection on the specific interaction between plasmonic nanoparticles and luminescent emitters. Through use of single-particle measurements, insightful works have recently been performed on luminescent lanthanide materials that are integrated with individual plasmonic nanoparticles.^34−37^ Nevertheless, these investigations mainly involve the upconversion luminescence of Er^3+^ with two major electric dipole emissions in the visible region. The more useful optical information and controllable multipeak manipulation for plasmon-enhanced down-shifting PL have been lacking. They are expected to be resolved with other RE ions.

Based on the above analysis, we designed (Au core)@(Y(V,P)O_4_:Eu) nanostructures and studied the selective PL emission enhancement of Eu^3+^ through the interaction with the plasmon resonance of the Au core. Gold nanospheres (NSs) of different diameters were employed as the cores. They give adjustable LSPR peaks. The Y(V,P)O_4_:Eu phosphor shell encapsulates entirely the Au NS core to form an isotropic core@shell nanostructure. The utilization of trivalent europium provides several advantages. First, Eu^3+^ possesses various emission lines that appear compactly in the visible region,^38^ which enables the selective emission enhancement easily. Second, Eu^3+^ usually shows a relatively high internal quantum yield in the down-shifting luminescence,^39^ which largely increases the spectral signal-to-noise ratio to give more reliable results. Third, the Eu^3+^ luminescence contains not only electric dipole but also magnetic dipole transitions,^33^ which largely enrich the interaction mechanism between the luminescent center and the plasmonic nanoparticle. Nevertheless, it is also important to clarify three issues in regard to the Eu^3+^ selective enhancement, including the comparison of the emission enhancement between different excitation states, such as ^5^D_0_ and ^5^D_1_, the effect of the plasmon resonance on the relaxation process from the ^5^D_1_ to ^5^D_0_ level, and the enhancement capabilities of the electric plasmon resonance for the electric and magnetic dipole transitions. In view of these considerations, the selective emission enhancement, expressed by the relative branching ratios (RBRs) for the five emission peaks of Eu^3+^, was analyzed in this work at the single-particle level through control of the effective LSPR–PL spectral overlap. The LIR modulation of the ^5^D_0,1_ → ^7^F_*J*_ transitions enables the designed materials to serve in multifunctional applications, including anticounterfeiting and optical temperature measurements during photothermal conversion.

## Results and Discussion

**Figure 1:**
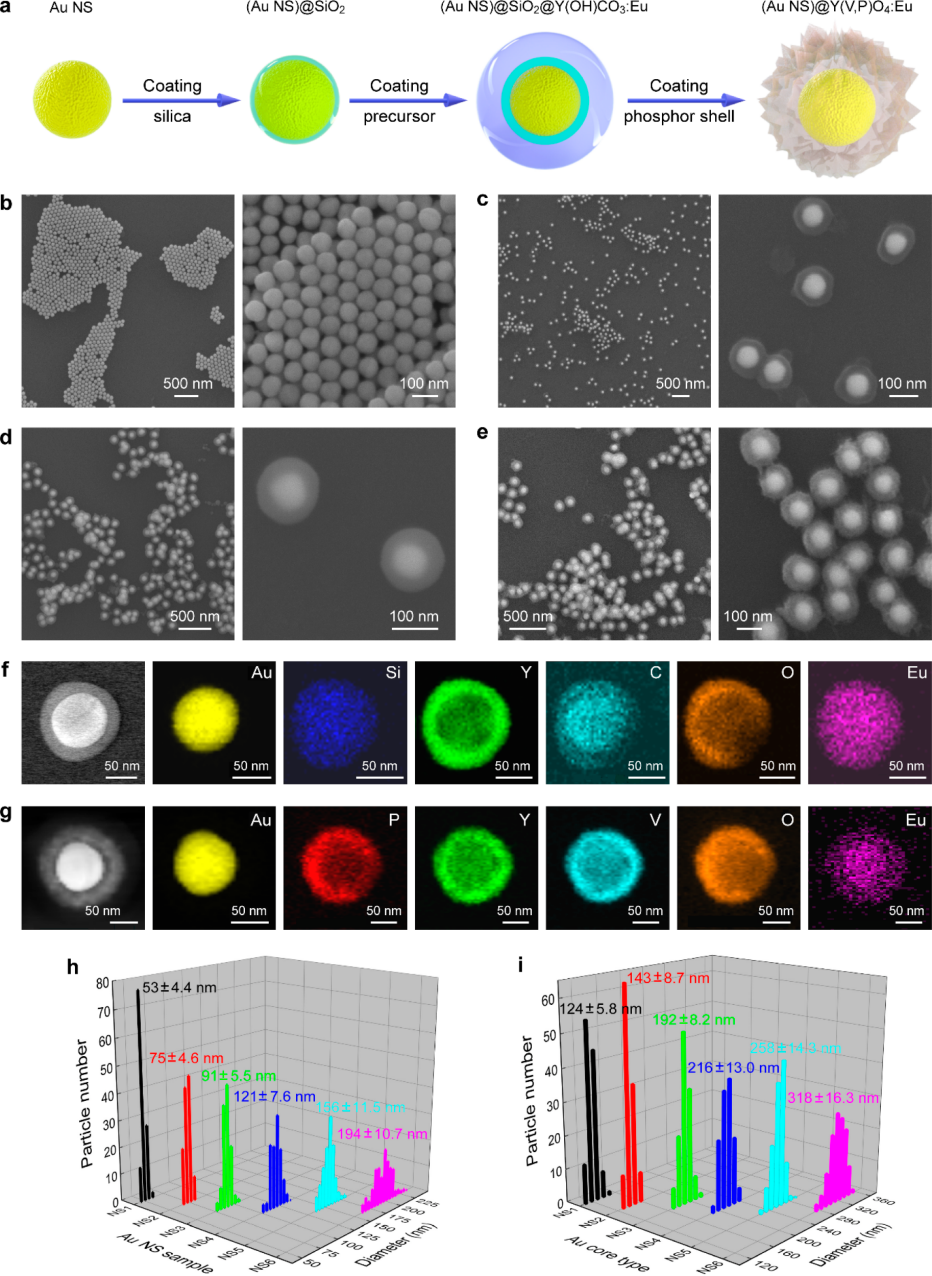
Preparation of the (Au core)@(Y(V,P)O_4_:Eu) nanostructures. (a) Schematic illustration of the synthesis route to the (Au NS)@Y(V,P)O_4_:Eu structure. The reactions include (i) the synthesis of the Au NSs, (ii) the coating of silica on the Au NSs by a modified Stöber method, (iii) the coating of Y(OH)CO_3_:Eu by a urea-based homogeneous precipitation process, and (iv) the hydrothermal reaction to give (Au NS)@Y(V,P)O_4_:Eu by a sacrificial-template method. (b–e) SEM images of the Au NS2 (b), (Au NS2)@SiO_2_ (c), (Au NS2)@SiO_2_@Y(OH)CO_3_:Eu (d), and (Au NS2)@Y(V,P)O_4_:Eu (e) at two different magnifications. (f and g) High-angle annular dark-field scanning transmission electron microscopy (HAADF-STEM) and elemental mapping images of the (Au NS2)@SiO_2_@Y(OH)CO_3_:Eu (f) and the (Au NS2)@Y(V,P)O_4_:Eu (g) nanostructures. (h and i) Particle size distributions of the Au NS samples (h) and the (Au NS)@Y(V,P)O_4_:Eu samples (i).

The wet-chemistry synthesis of the core@shell nanostructures involves four steps, as illustrated schematically in Figure 1a. Six Au NS samples of various diameters are first prepared by seed-mediated growth,^40^ with the synthesis details given in the Supporting Information. They are labeled as Au NS1–6, and their average diameters are 53 ± 4.4, 75 ± 4.6, 91 ± 5.5, 121 ± 7.6, 156 ± 11.5, and 194 ± 10.7 nm, respectively. A homogeneous silica shell is subsequently coated on the CTAB-stabilized Au NSs following a modified Stöber method.^30,41,42^ In the third step, a phosphor precursor Y(OH)CO_3_:Eu as the outmost shell is grown on the SiO_2_ shell surface by a urea-based homogeneous precipitation process.^43,44^ Finally, the (Au NS)@Y(V,P)O_4_:Eu product is obtained hydrothermally by use of the Y(OH)CO_3_:Eu shell as a sacrificial template. At the same time, the SiO_2_ layer is almost completely dissolved in the hydrothermal reaction, as confirmed below. The morphological characteristics were checked by scanning/transmission electron microscopy (SEM/TEM) imaging. Parts b–e of Figure 1 show the SEM images at the different synthetic stages for the representative end product (Au NS2)@Y(V,P)O_4_:Eu. The nanoparticles obtained at the different stages possess good size uniformity. All the shells, including SiO_2_, Y(OH)CO_3_:Eu, and Y(V,P)O_4_:Eu, encapsulate the corresponding Au cores tightly and completely. As a result, the nearly isotropically spherical core@shell nanostructures can effectively remove the particle-orientation disturbance in the spectral measurements. The morphological details of these core@shell nanostructures can be further observed from their TEM images (Figure S1). The Y(V,P)O_4_:Eu shell is seen to be composed of many small nanocrystals, which are tightly aggregated around the Au cores. The as-prepared intermediate and final samples based on the other Au NS samples of different sizes exhibit similar uniformities in morphology and size (Figures S2–S6).

Parts f and g of Figure 1 display the high-angle annular dark-field scanning transmission electron microscopy (HAADF-STEM) and elemental mapping images of the representative (Au NS2)@SiO_2_@Y(OH)CO_3_:Eu and (Au NS2)@Y(V,P)O_4_:Eu nanostructures, respectively. The results for two other representative (Au NS4, NS6)@Y(V,P)O_4_:Eu samples are displayed in Figure S7. The Au atoms were found to be located only at the cores for both spherical particles. The other elements are distributed throughout the shell area to form uniform chemical phases. It is worth pointing out that the Si signal was hardly observed in the elemental mapping of (Au NS2)@Y(V,P)O_4_:Eu, implying that the SiO_2_ shell is almost completely etched away in the hydrothermal process under the hydrothermal conditions. This view is further supported by the energy-dispersive X-ray (EDX) analysis (Figure S8). The EDX spectra reveal that all the constituent elements in the above two core@shell samples are detectable and that no other impurity elements are present. However, the relative atomic ratio of Si in (Au NS2)@Y(V,P)O_4_:Eu is reduced more than 10-fold in comparison to that in (Au NS2)@SiO_2_@Y(OH)CO_3_:Eu. The Si content is therefore negligible in the (Au NS2)@Y(V,P)O_4_:Eu nanostructures, although there is a considerably thick SiO_2_ shell in the (Au NS2)@SiO_2_ intermediate product (Figure S9).

Parts h and i of Figure 1 show the particle size distributions of the six Au NS samples and the finally obtained (Au NS)@Y(V,P)O_4_:Eu samples, respectively. From NS1 to NS6, not only the uncoated Au cores but also the (Au NS)@Y(V,P)O_4_:Eu nanostructures exhibit increasing average diameters (Figure S10), accompanied by growing shell thicknesses that range approximately from 36 to 62 nm. Previous studies have indicated that the fluorescence quenching of an emitter becomes dominant when the spacing between a plasmonic metal nanoparticle and the emitter is within 5 nm, where the nonradiative decay rate follows a *R*^–6^ dependence, with *R* being the distance between the emitter and the center of the plasmonic nanoparticle.^45−47^ As a result, a spacing larger than 5 nm is generally beneficial to the PL enhancement. In addition, effective emission enhancement has also been witnessed in many luminescent materials when the spacing is varied in a wide range of 5–50 nm.^21,22,28,30,48^ Therefore, the Y(V,P)O_4_:Eu shell with a thickness of 36–62 nm provides a suitable distance between the plasmonic Au nanoparticle and the Eu^3+^ emitters in the outer shell for the utilization of the desired PL enhancement.

**Figure 2:**
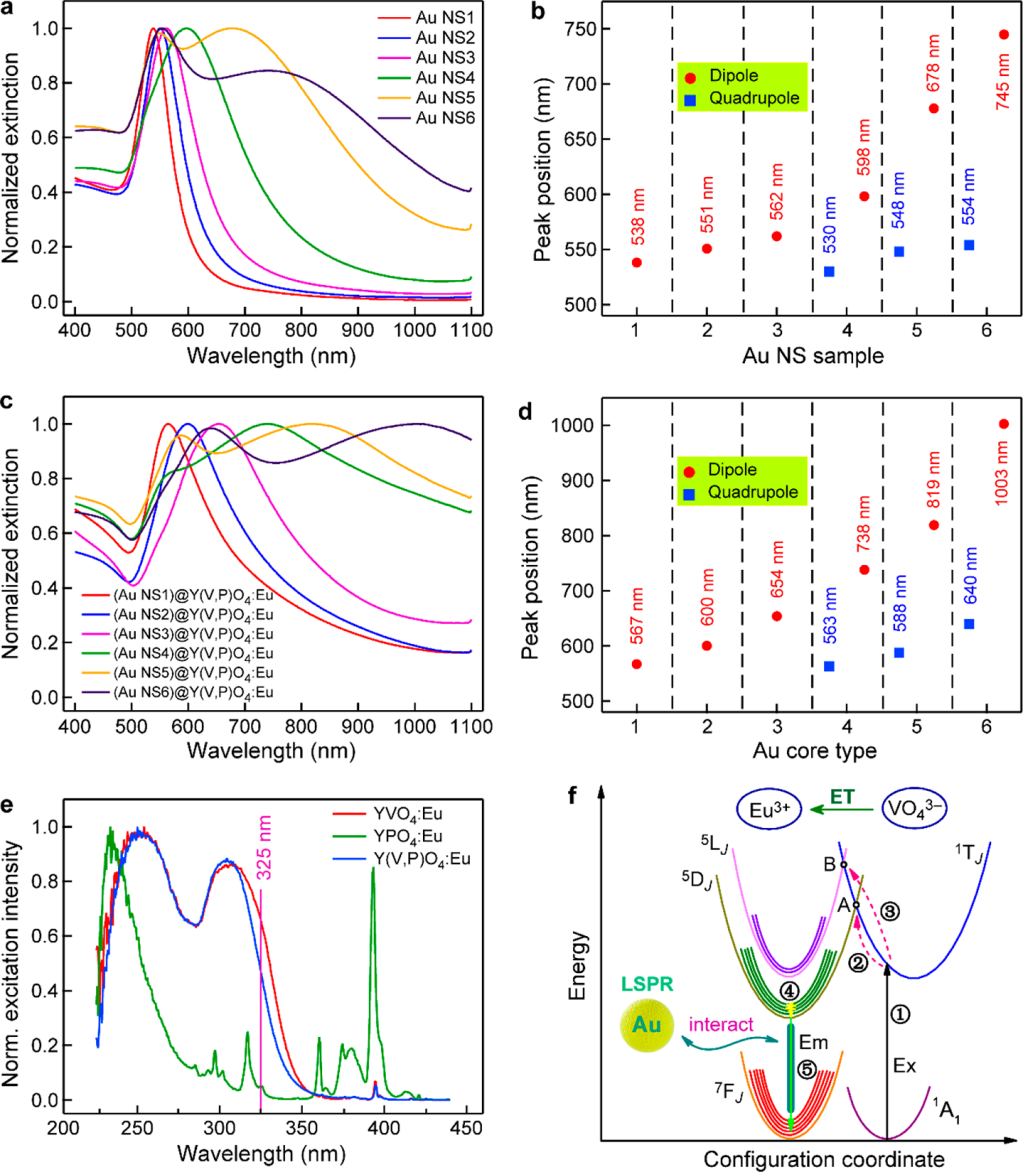
Extinction and excitation spectra of the core@shell samples. (a) Extinction spectra of the differently sized Au NS samples. (b) Extinction peak positions of the Au NS samples. Two extinction peaks are present. They are the dipole and quadrupole plasmon resonance modes. (c) Extinction spectra of the six (Au NS)@Y(V,P)O_4_:Eu samples. (d) Extinction peak positions of the six (Au NS)@Y(V,P)O_4_:Eu samples. (e) Normalized excitation spectra of the YVO_4_:Eu, YPO_4_:Eu, and Y(V,P)O_4_:Eu samples. The PL detection wavelength is 617 nm for the three samples. (f) Configuration coordinate diagram for VO_4_^3–^ and Eu^3+^ to show the ET process between them and schematic illustrating the emission enhancement by the plasmon resonance.

Figure 2a presents the extinction spectra of the six differently sized Au NS samples. With increasing NS diameters, the plasmon peak becomes broadened, which is attributed to the increasing radiative damping at larger sizes.^40,49^ In addition, the relatively small Au NSs possess only one extinction peak, which should arise from the dipole plasmon mode. A shoulder extinction peak at the higher-energy side emerges with the average Au diameter growing to 156 nm. This peak even becomes dominant when the average diameter reaches 194 nm. This emerging extinction peak is ascribed to the quadrupole plasmon mode, as verified in the simulation below. Figure 2b reveals the extinction peak positions of the six Au NS samples. A red shift phenomenon with increasing diameters is clearly visible for both plasmon modes. When the Au NSs are encapsulated with the Y(V,P)O_4_:Eu shell, a clear spectral broadening occurs for every (Au NS)@Y(V,P)O_4_:Eu nanostructure sample (Figure 2c), accompanied by clear red shifts relative to the peaks of the corresponding bare Au NS samples (Figure 2b,d). These observations stem mainly from the increasing size distributions of the core@shell nanostructures and the increased refractive index around the Au NSs.^50^

To ascertain the light absorption properties of the core@shell samples and explain the use of the solid solution in the Y(V,P)O_4_:Eu shell, the normalized excitation spectra of the YVO_4_:Eu, YPO_4_:Eu, and Y(V,P)O_4_:Eu samples are provided in Figure 2e, where the Eu contents were adjusted to be close to that in the core@shell samples. The PL excitation wavelength used in our study was 325 nm. The very low excitation intensity at 325 nm for YPO_4_:Eu makes single-particle PL measurements unsuitable. A broader and stronger excitation band in the region around 325 nm is achieved from the charge-transfer absorption of the VO_4_^3–^ group in YVO_4_:Eu.^51,52^ However, the difficulty in the synthesis of homogeneous core@shell nanostructures using YVO_4_:Eu as the luminescent shell prevents it from further investigation (Figure S11). The morphological inhomogeneity makes it difficult to determine the distance between the plamonic Au core and the phosphor shell for (Au NS)@YVO_4_:Eu. Fortunately, the solid solution method by partially replacing V with P (15 mol % in this work) to form Y(V,P)O_4_:Eu overcame the problems mentioned above. The introduction of P caused the reaction with the Y(OH)CO_3_:Eu precursor to produce many small nanocrystals (Figure S12). These nanocrystals were further assembled as a compact homogeneous spherical shell around the Au NS cores in the coexistence of V and P. The X-ray diffraction (XRD) patterns (Figure S13) indicate the successful preparation of the solid solution structure.

Although the introduction of P into YVO_4_:Eu leads to the partial sacrifice of the excitation intensity at 325 nm (Figure 2e), the efficient ET from the VO_4_^3–^ group to Eu^3+^ can still occur. As depicted in Figure 2f, the VO_4_^3–^ group is first excited with the 325 nm laser (Process 1). The energy is then transferred to Eu^3+^, populating the ^5^L_*J*_ or ^5^D_*J*_ levels (Processes 2 and 3).^51^ After relaxation (Process 4), the electron returns to the ^7^F_*J*_ ground states, creating the visible emissions (Process 5). If the luminescent ion is suitably positioned around a plasmonic nanostructure, its PL process will be further modified. To clearly understand the effect of the plasmon resonance, the apparent PL intensity (*I*) of a plasmon-enhanced Eu^3+^emission can be described by the following equation^53,54^
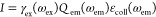
1where γ_ex_(ω_ex_) is the near-field excitation rate of the luminescent ion at the excitation frequency ω_ex_, *Q*_em_(ω_em_) is the quantum yield for the far-field emission at the emission frequency ω_em_, and ε_coll_(ω_em_) is the light-collection efficiency of the optical measurement system. On the other hand, in the present investigation, we just compare the relative PL intensities of the different emission peaks that originate from the same activator (Eu^3+^) by use of the fixed excitation wavelength at 325 nm. The different emission peaks share the same excitation path into the energy level of the VO_4_^3–^ group. As a result, γ_ex_(ω_ex_) can be dropped out in the analysis of the plasmonic enhancement for the different emission peaks of Eu^3+^. We will therefore consider only *Q*_em_(ω_em_) to simplify the analysis. As indicated in Figure 2f, the introduction of plasmon resonance can lead to a clear emission enhancement because of the large confinement and enhancement of the electromagnetic field.

**Figure 3:**
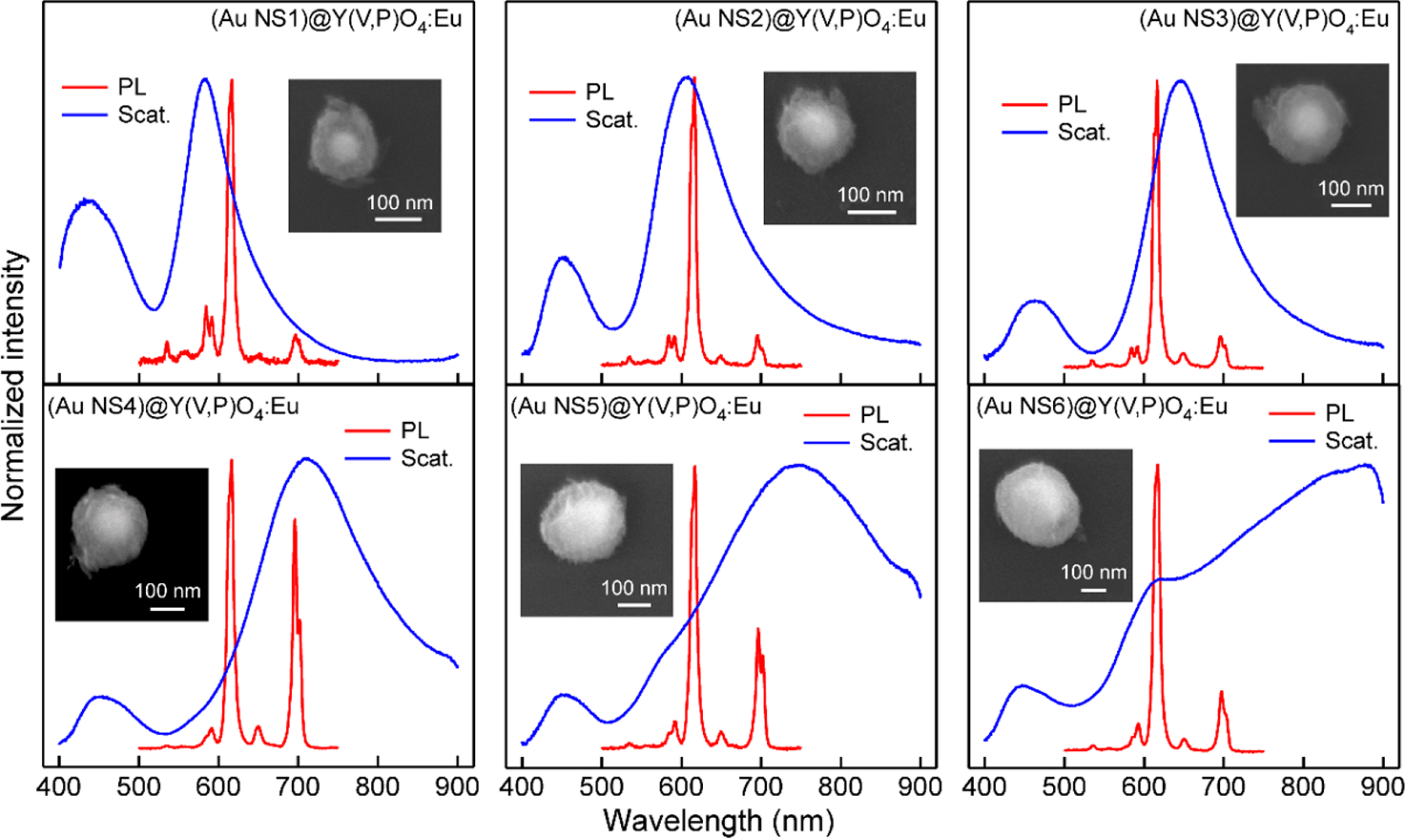
Scattering and PL spectra of the different core@shell nanostructures. The spectra of a representative (Au NS)@Y(V,P)O_4_:Eu nanostructure is given for each Au NS sample. The insets show the SEM images of the corresponding core@shell nanostructures.

The single-particle scattering and PL measurements are important for understanding plasmon-enhanced emissions. Silicon and indium–tin oxide (ITO)-coated glass substrates are two types of widely used conductive substrates for these measurements. Although the latter has a lower refractive index to minimize the interaction between plasmonic nanoparticles and the substrate, strong PL signals are generated on it under laser excitation at 325 nm,^55^ which causes difficulty in analyzing the PL spectra of the core@shell nanostructures. Silicon possesses a larger refractive index, but it does not induce any clear changes in the scattering spectrum and peak wavelength in comparison with the ITO substrate (Figure S14). This can be ascribed to the thick spacer layer of the phosphor shell between the Au NSs and the silicon substrate. We therefore employed silicon as the substrate in the spectral measurements on the individual (Au NS)@Y(V,P)O_4_:Eu nanostructures. The correlation between dark-field and SEM imaging was realized by a pattern-matching method.^56^Figure 3 displays the representative normalized scattering and PL spectra of the individual nanostructures from the six core@shell samples in conjunction with the SEM images of the corresponding core@shell nanostructures. For any scattering spectrum regardless of the particle size, a relatively weak scattering peak before 500 nm was observed. Its intensity gets weaker in comparison with the dominant peak when the particle is increased in size. This weak scattering peak was confirmed to arise from the Y(V,P)O_4_:Eu shell through comparison with the scattering spectra of the individual Au-free phosphor spheres, which exhibit a scattering peak at the similar spectral position (Figure S15). On the other hand, the dominant scattering peaks for the differently sized particles are all originated from the Au NSs. The shoulder scattering peaks also appear for the larger particles. They are similar to those observed in the extinction spectra (Figure 2c). To ascertain the origins of the scattering peaks, finite-difference time-domain (FDTD) simulations were performed, where the size parameters of the nanostructures were determined from the SEM images (Figure S16). The average refractive index of YVO_4_ is above 2.0 in the spectral range 500–900 nm.^57^ It should be adjusted in our study because the Y(V,P)O_4_:Eu shell is made of nanocrystal aggregates and contains a small amount of phosphorus. We determined the refractive index of Y(V,P)O_4_:Eu by matching the FDTD-simulated scattering spectra with the measured ones. A representative core@shell structure was chosen from each (Au NS)@Y(V,P)O_4_:Eu sample. Their scattering spectra were simulated separately. The results reveal that all the simulated scattering spectra can match well with the measured ones when a refractive index of 1.76 was used for the phosphor shell. The nature of the plasmon resonance modes for the different scattering peaks in Figure 2d were also determined from the simulations (Figure S17). The strong LSPR peak for the small particles is originated from the dipole plasmon resonance, whereas the emerging shoulder peak for the large particles can be ascribed to the quadrupole plasmon resonance.

In a previous work,^58^ the optimal Eu^3+^-doping concentration was determined to be 5 mol % in bulk YVO_4_ material. The same Eu^3+^ content was therefore used in this work. For the PL spectra in Figure 3, several line emissions were observed. The dominant one is located at 617 nm, which is attributed to the ^5^D_0_ → ^7^F_2_ transition of Eu^3+^. The weak emissions at 650 and 697 nm are ascribed to the ^5^D_0_ → ^7^F_3_ and ^5^D_0_ → ^7^F_4_ transitions of Eu^3+^, respectively.^44^ It is interesting to see two partially overlapping emission peaks in the region 570–599 nm. To find out if the two emission peaks belong to the same dipole transition, the temperature-dependent PL spectra were analyzed (Figure S18). When the spectra are normalized at 617 nm, the emission peaks at 592 nm can overlap completely with each other, but those at 584 nm exhibit gradual enhancement with rising temperatures. This observation implies that the 592 nm emissions can be attributed to the ^5^D_0_ → ^7^F_1_ transition of Eu^3+^, while the 584 nm ones should be originated from another excited state. According to a previous study,^38^ this emission peak can be assigned to the ^5^D_1_ → ^7^F_3_ transition of Eu^3+^. Moreover, the weak sharp peak appearing at 536 nm can be ascribed to the ^5^D_1_ → ^7^F_1_ transition of Eu^3+^. These attributions were further confirmed by the transient decay kinetics (Figure S19 and Table S1). Very close decay lifetime values were obtained when the emissions were monitored at 536 and 584 nm. They are far from that obtained by monitoring the emissions at 592 nm. This result implies that the 536 and 584 nm emission peaks are produced from the same excited state, but different from the 592 nm emission peak. In this case, the two adjacent peaks from 570 to 599 nm should be separated to correlate their individual PL enhancement with the plasmon resonance. Gaussian fitting was employed to distinguish the two emission peaks at 584 and 592 nm (Figure S20). The 584 nm emission peak is first weakened gradually with increasing particle diameters and becomes much weaker than the 592 nm emission peak for the large core@shell nanostructures. In addition, the other emission peaks of Eu^3+^ in Figure 3 also vary in intensity as functions of the particle size and thus the scattering wavelength. In general, the PL enhancement of one particular emission peak of Eu^3+^ is maximized when the plasmonic scattering peak overlaps closely with the emission peak. To ascertain more clearly the relationship between the PL enhancement and the plasmon wavelength, statistical analysis was further performed.

**Figure 4:**
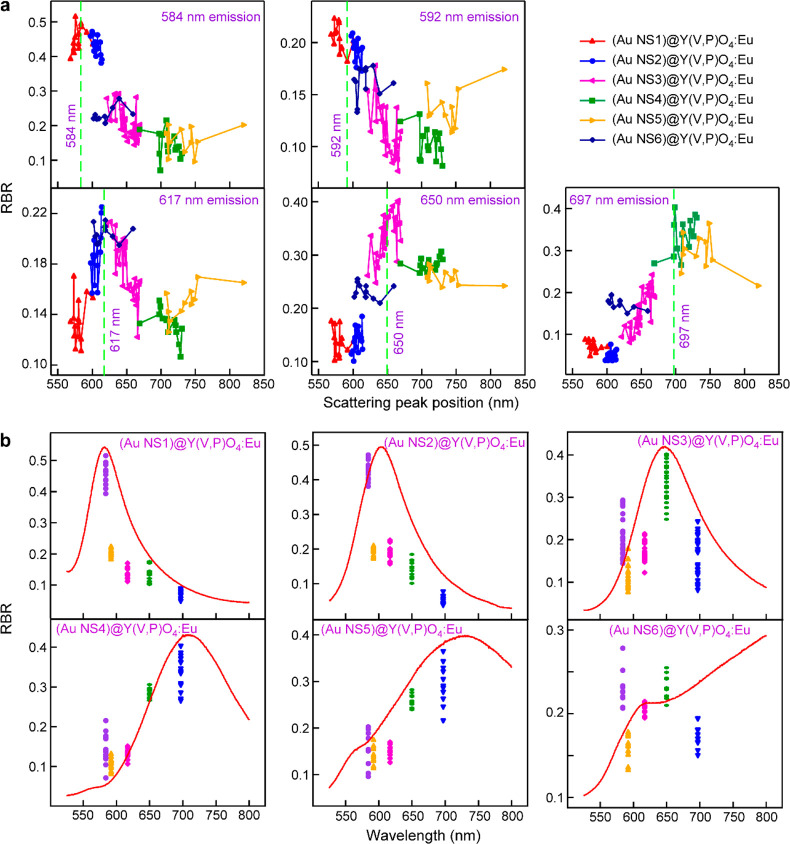
Relationship between the emission enhancement and the LSPR peak. (a) Dependences of the RBR values on the scattering peak wavelength for the individual (Au NS)@Y(V,P)O_4_:Eu nanostructures. Five emission peaks of Eu^3+^ were considered. The scattering peaks resulting from the dipole plasmon mode were used for (Au NS1–NS5)@Y(V,P)O_4_:Eu, and those arising from the quadrupole plasmon mode for (Au NS6)Y(V,P)O_4_:Eu were used. The plots are derived from the single-particle spectral measurements on 14, 15, 27, 14, 11, and 12 individual particles for (Au NS1–NS6)@Y(V,P)O_4_:Eu, respectively. (b) RBR values of the five emission peaks of Eu^3+^ and representative scattering spectra (red curves) for the six (Au NS1–NS6)@Y(V,P)O_4_:Eu samples. The RBR data points are the same as those in (a). The six scattering spectra are normalized.

A total of 93 individual differently sized (Au NS)@Y(V,P)O_4_:Eu nanostructures were randomly selected to uncover statistically the relationship between the selectively enhanced emissions and the LSPR peaks. To characterize the PL enhancement behaviors quantitatively, the RBRs of different emission peaks are often utilized.^33,55^ The definition of the RBR for a particular emission of Eu^3+^ is expressed as the relative weight of its integrated intensity that is normalized against the intrinsic oscillator strength of the corresponding dipole transition moment,^55^ as shown in eq 2
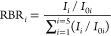
2where *I*_*i*_ is the integrated PL intensity of the *i*-th emission peak of the individual (Au NS)@Y(V,P)O_4_:Eu nanostructure and *I*_0*i*_ is the integrated PL intensity of the *i*-th emission peak of the bulk Y(V,P)O_4_:Eu sample. The intrinsic oscillator strengths were acquired from the PL spectrum of a gold-free bulk Y(V,P)O_4_:Eu sample (Figure S21). The five emissions, including one ^5^D_1_ → ^7^F_3_ (584 nm) and four ^5^D_0_ → ^7^F_1–4_ (592, 617, 650, and 697 nm) transitions, were statistically analyzed. They are denoted with the *i*-th emission peak in sequence (*i* = 1–5). The integration wavelength range for the 584 and 592 nm emission peaks is 570–599 nm, where Gaussian fitting was applied. The integration wavelength ranges are 600–630, 640–665, and 680–710 nm for the 617, 650, and 697 nm emission peaks, respectively. Figure 4a displays the dependences of the RBRs for the five Eu^3+^ emission peaks on the plasmonic scattering peak position of (Au NS1–NS6)@Y(V,P)O_4_:Eu. The data points were extracted from the single-particle scattering and PL spectra of the six core@shell nanostructure samples. The peak position of the dipole plasmon resonance mode of (Au NS6)@Y(V,P)O_4_:Eu is beyond 800 nm and spectrally away from all the emission peaks of Eu^3+^. The quadrupole plasmon peak was therefore used in the analysis. The clear variation trends of the RBRs against the plasmon wavelength suggest the strong interplay between PL and LSPR. Specifically, the shift of the plasmon peak near an emission peak maximizes the RBR of that emission peak. Based on eq 1, the emission intensity of a particular transition dipole (*I*_*i*_) can be described as^54^
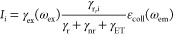
3where γ_r,*i*_ is the radiative decay rate of the considered emission peak, γ_r_ and γ_nr_ are the total radiative and nonradiative decay rates of the emitter, respectively, and γ_ET_ is the ET rate from the emitter to the plasmonic nanoparticle. If we adjust appropriately the spectral overlap between the LSPR and PL peaks as well as the spacing between the plasmonic nanoparticle and the luminescent center, a significant increase of the radiative decay rate γ_r,*i*_ can be expected due to the largely local density of photonic states. As a result, the selective emission enhancement of Eu^3+^ is realized just by modulating the LSPR wavelength, as presented in Figure 4a.

A careful look at the plasmon-wavelength-dependent RBRs for the 584, 592, and 617 nm three emission peaks in Figure 4a led to the observation that the RBRs are gradually increased in the order from (Au NS4)@Y(V,P)O_4_:Eu to (Au NS6)@Y(V,P)O_4_:Eu, with the last sample exhibiting a particularly significant RBR increase in contrast with the former two samples. This result is mainly caused by the quadrupole plasmon resonance of the largest Au NSs, the scattering peak of which exactly overlaps with the three emission peaks and therefore results in their selective PL enhancement. On the other hand, the ^5^D_0_ → ^7^F_1_ emission (592 nm), arising from a magnetic dipole transition, is also enhanced to some degree by the electric plasmon resonance of the Au NSs, as indicated by the variation trend of the RBRs with the scattering wavelength. However, the relatively low RBR values suggest a weaker interplay between the magnetic dipole transition and the electric plasmon resonance. This result is further supported by the relations among the RBRs, the emission wavelengths, and the plasmon peak positions, as presented statistically from another perspective (Figure 4b). The comparison of the RBRs for the different emission peaks is clearly seen for every core@shell nanostructure sample. When the Au core changes from NS1 to NS2, the RBRs of the 617 nm emission peak increase considerably in comparison with that of the 592 nm emission peak despite the fact that the two emission peaks overlap spectrally with the LSPR peak to a similar degree. This observation can be ascribed to the stronger interaction between electric dipole transitions and an electric plasmon resonance than that between magnetic dipole transitions and an electric plasmon resonance.^55^ Further evidence for this observation can be traced from the ratio changes of the PL EFs for different emission peaks. The ratio can be expressed as follows according to eq 2
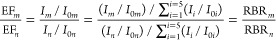
4where the subscripts *m* and *n* denote, respectively, the *m*- and *n*-th emission peaks of Eu^3+^, *I*_*i*_ (or *I*_*m*_, *I*_*n*_) is the integrated intensity of the *i*-th (or *m*-th, *n*-th) emission peak of the individual (Au NS)@Y(V,P)O_4_:Eu nanostructure, and *I*_0*i*_ (or *I*_0*m*_, *I*_0*n*_) is the integrated intensity of the *i*-th (or *m*-th, *n*-th) emission peak of the bulk Y(V,P)O_4_:Eu sample. The 14 individual (Au NS1)@Y(V,P)O_4_:Eu nanostructures were all considered (Figure S22a). On the one hand, the EF_592_/EF_697_ value exhibits an overall upward trend as the plasmon peak is red-shifted. EF_697_ is believed to remain nearly unchanged because the 697 nm emission peak is far away from the plasmon peak. As a result, a rising tendency for EF_592_ when the plasmon peak gradually approaches 592 nm implies that the magnetic dipole transitions are enhanced by the electric plasmon resonance. On the other hand, EF_584_/EF_592_ shows an increasing trend with the red-shifting plasmon peak. In particular, the emission intensity of the 584 nm peak exceeds that of the 592 nm peak even although the LSPR peak perfectly covers the 592 nm emission peak, as revealed by the scattering and PL results acquired from two (Au NS1)@Y(V,P)O_4_:Eu nanostructures (Figure S22b). These results can be partially ascribed to the more significant PL enhancement for the electric dipole transitions by the electric plasmon resonance. Another reason is related to the internal quantum yields of the emission peaks. Figure 4b shows that the average RBR for the 584 nm peak is predominant for all core@shell nanostructure samples among the three neighboring 584, 592, and 617 nm emission peaks even if the 584 nm emission peak is far away from the LSPR peak than the other two for the larger nanostructures. Previous investigations suggest that a lower intrinsic quantum yield of fluorophores brings a larger enhancement in the overall quantum yield.^54,59^ Different from common fluorophores, Eu^3+^ possesses five emission peaks in the core@shell nanostructures. Their respective PL enhancements with the adjustable LSPR peaks should be treated separately. The internal quantum yield (η) is defined as the ratio of the emitted photon number (*P*_e_) to the absorbed photon number (*P*_a_). It can be converted to the expressions of the emission intensity (*I*_e_) and the absorption intensity (*I*_a_) as follows
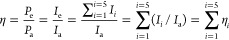
5where *I*_*i*_ and η_*i*_ are the integrated intensity and the internal quantum yield of the *i*-th emission peak of Eu^3+^ in the individual (Au NS)@Y(V,P)O_4_:Eu nanostructure, respectively. η_*i*_ can be further written as
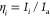
6

Since all the emission peaks of Eu^3+^ share the same denominator, a weaker intrinsic transition should correspond to a lower intrinsic quantum yield. When the LSPR peak overlaps well with the emission peak, the weakest intrinsic emissions at 584 nm (Figure S21) enable the most significant PL enhancement.

The above quantitative statistical analysis of the relationship of the RBRs with the LSPR and PL peaks indicates that the effective spectral overlap between the plasmon and the emission peaks and the intrinsic quantum yield of the luminescent center jointly contribute to the magnitude of the selective emission enhancement. To investigate the plasmonic effect on the dynamics of the PL emissions, we further measured the luminescence lifetimes of Eu^3+^ on the different excitation states (Figure S23 and Tables S2–S4). The dramatic decreases of the decay lifetime values for the ^5^D_0_ level upon the introduction of the Au NS core suggest strong coupling between the plasmonic Au NS core and the phosphor shell, which introduces new decay pathways to the luminescence processes. The decay lifetime values measured by monitoring the emissions at 617 and 697 nm display similar changes with the particle diameter. Both reach their minima for the Au NS2 cores whose LSPR peak overlaps closely with the ^5^D_0_ → ^7^F_2_ transition (617 nm). This is because the integrated intensity of this emission peak is much larger than those of the other four. The interaction between the plasmon resonance and the Eu^3+5^D_0_ → ^7^F_2_ transition accelerates the de-excitation rate from the ^5^D_0_ excitation state. In regard to the ^5^D_1_ excitation state, both the 536 and 584 nm emission peaks are involved (Figure S19). The 536 nm peak rather than the 584 nm one was used for monitoring to characterize the decay rate of the ^5^D_1_ level in order to minimize the spectral interference from the 592 nm emission peak. Interestingly, the decay lifetime minimum was observed also for (Au NS2)@Y(V,P)O_4_:Eu instead of (Au NS1)@Y(V,P)O_4_:Eu although the LSPR peak of the latter is closer to the spectral range of the ^5^D_1_ → ^7^F_*J*_ transitions. A possible reason is connected to the intensified relaxation rate to the ^5^D_0_ level from the upper level (Process 4 in Figure 2f). The relaxation rate to the ^5^D_0_ level is probably largely enhanced by the sharply increased de-excitation rate of the ^5^D_0_ level when the plasmon peak overlaps with the 617 nm emission peak.

The combination of LSPR and RE-based luminescence emissions offers many opportunities for developing applications in different fields. We employed our (Au NS)@Y(V,P)O_4_:Eu core@shell samples in two applications, which are anticounterfeiting and optical temperature measurements for photothermal conversion on the basis of the selective Eu^3+^ emission enhancement by the plasmon resonance of the Au NS core. Over the past decades, various security technologies have been developed to combat counterfeiting, ranging from simple watermarks and metal threads to spectroscopic analysis.^60−62^ In particular, luminescence-induced functional materials based on colorimetric and fluorometric detection designs for authentication have received much attention.^63,64^ Nevertheless, with fast development and generalization of high-resolution equipment, counterfeiters are able to produce high-quality counterfeit products. Advanced anticounterfeiting has thus become ever highly desired. In this work, we provide a bimode optical anticounterfeiting strategy, employing the interaction between the plasmon resonance and the PL signal to obtain characteristic scattering color and LIR.

**Figure 5:**
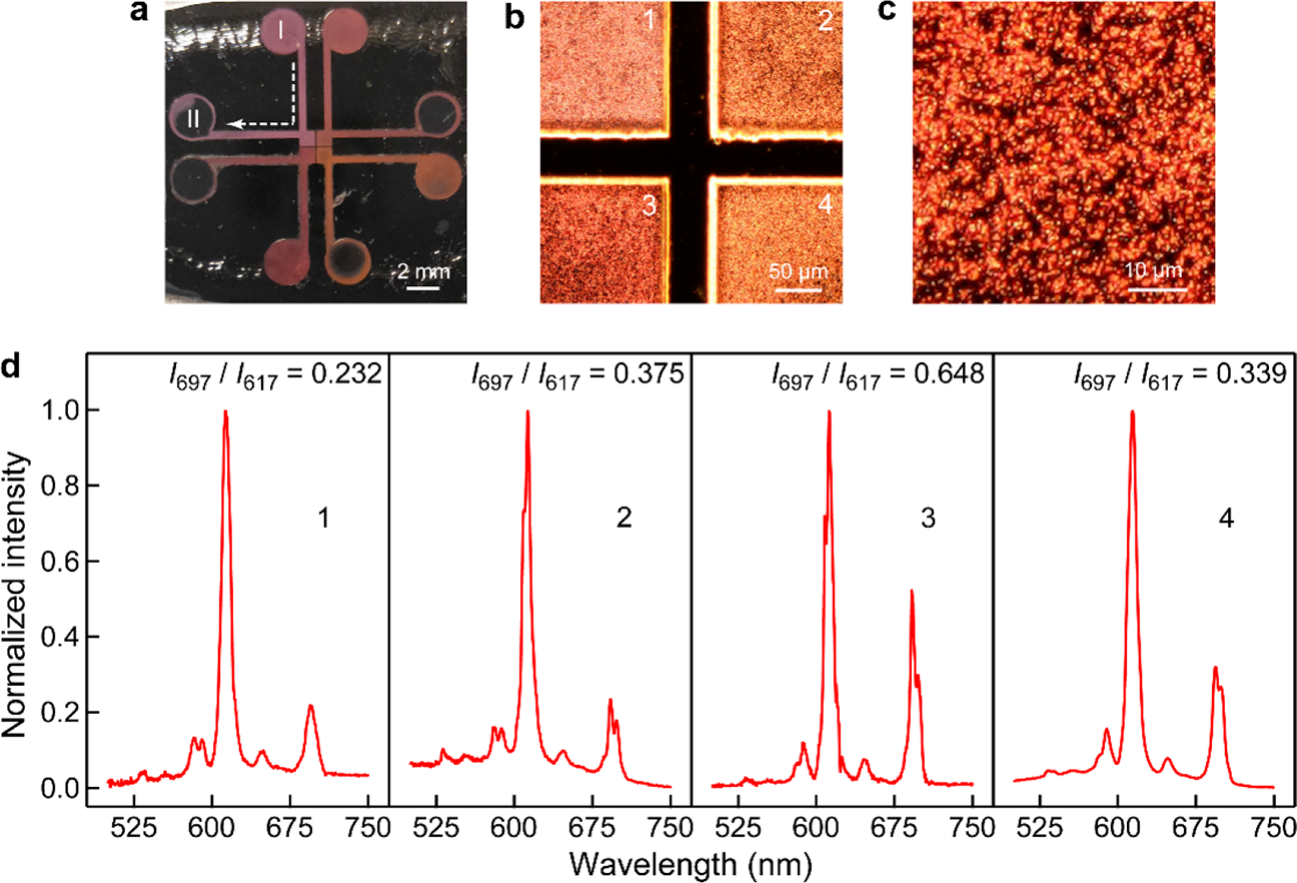
Anticounterfeiting devices. (a) Fabricated square device with different samples under daylight. The device is composed of four square quadrants at the center. For each square, the suspension liquid was injected from the circular groove at one end (marked with I). The suspension liquid subsequently flowed through the straight microfluidic channel and reached the square groove at the center. After the square groove was filled up, the leftover suspension liquid flowed further through the straight microfluidic channel to the circular groove at the other end (marked with II). (b) Optical image of the four square quadrants at the center of the device. The luminescent materials used in squares 1–4 are the (Au NS1, NS2, NS4, NS6)@Y(V,P)O_4_:Eu samples, respectively. (c) Optical image of square 3 in (b) at higher magnification. (d) PL spectra measured at the four square quadrants in the fabricated device. The excitation wavelength was 325 nm.

Figure 5a shows the fabricated square device, which used the (Au NS1, NS2, NS4, NS6)@Y(V,P)O_4_:Eu samples for anticounterfeiting. The four square quadrants at the center appear similarly dark pink under daylight. Their far-field scattering patterns (Figure 5b) show that quadrant 3 exhibits a darker salmon-pink color than the other three, as can be understood from Figure S24. Bright dots are seen on the scattering patterns at higher magnification (Figure 5c). They correspond to the individual (Au NS)@Y(V,P)O_4_:Eu nanoparticles. In comparison with the similar square device made from the related luminescent materials regardless of the particle size (Figure S25a–c), very bright and distinguishable colors were observed on the far-field scattering patterns only for the core@shell nanostructure samples. The vivid colors are attributed to the plasmon resonance peak of the Au NSs in the crimson region. The bright scattering colors of the (Au NS)@Y(V,P)O_4_:Eu nanostructure samples endow the fabricated device with the first feature for use in anticounterfeiting. Another feature for anticounterfeiting is luminescence. Similar to the single-particle PL spectral analysis, the device region filled with (Au NS4)@Y(V,P)O_4_:Eu exhibits a much larger LIR for 697 and 617 nm emission peaks than those filled with the other core@shell nanostructure samples (Figure 5d) and the Au-free phosphor samples (Figure S25d). The dual characteristics enable the (Au NS)@Y(V,P)O_4_:Eu nanostructures to be a promising candidate for high-performance anticounterfeiting applications.

**Figure 6:**
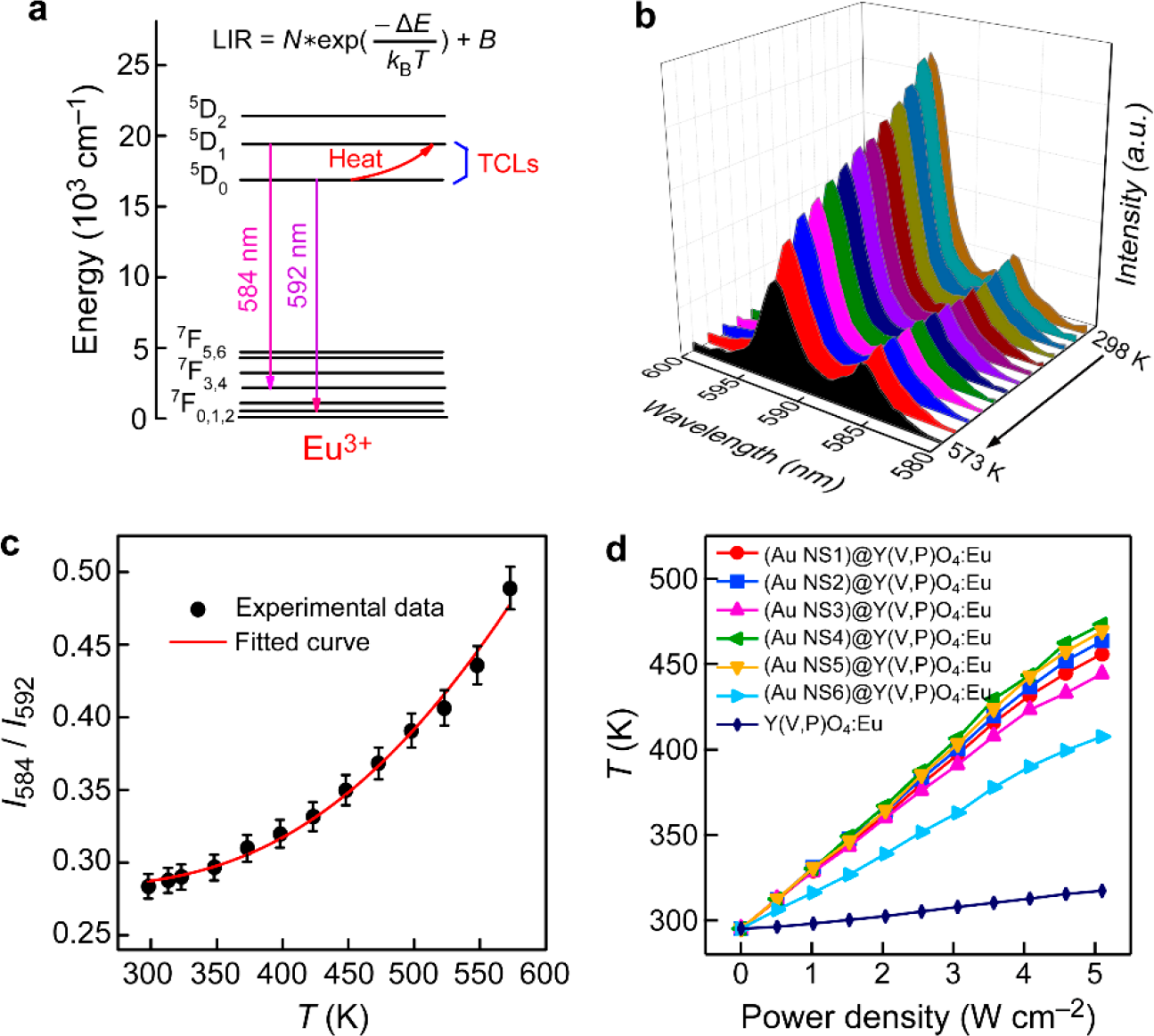
Optical temperature measurements during photothermal conversion. (a) Energy level diagram of Eu^3+^, showing the emissions related to the two TCLs. (b) PL spectra of (Au NS4)@Y(V,P)O_4_:Eu at various temperatures. (c) Dependence of *I*_584_/*I*_592_ on the absolute temperature. (d) Temperature traces of the different powder samples induced by plasmonic photothermal conversion as functions of the optical power densities at 808 nm from a diode laser.

The actual temperature of the (Au NS)@Y(V,P)O_4_:Eu powder samples induced by plasmonic photothermal conversion can be measured by the Eu^3+^-activated phosphor. The energy level diagram of Eu^3+^ (Figure 6a) illustrates the mechanism for the temperature measurement. Two thermally coupled levels (TCLs) of Eu^3+^ (^5^D_0_ and ^5^D_1_) are employed to produce the 584 and 592 nm emissions, the LIR of which follows the Boltzmann distribution^65^
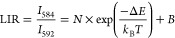
7where *N* and *B* are constants, Δ*E* is the energy gap between the TCLs, *k*_B_ is the Boltzmann constant, and *T* is the absolute temperature. The temperature-dependent PL spectra of the representative (Au NS4)@Y(V,P)O_4_:Eu sample reveal the different intensity variations for the two emission lines (Figure 6b and Figure S26). In particular, the 584 nm emission peak becomes stronger with increasing temperatures, while the 592 nm emission peak decreases in intensity. This phenomenon can mainly be attributed to the thermally coupled effect, which causes extra population on the upper ^5^D_1_ level as the temperature is raised. This extra population was also supported by the increasing decay lifetime for this excitation state (Figure S27 and Table S5). According to eq 7, the experimental data were well fitted according to  (Figure 6c), generating Δ*E* = 1583.1 cm^–1^. This value is near the reported energy gap (∼1700 cm^–1^) between ^5^D_0_ and ^5^D_1_ of the Eu^3+^ ion.^66^ In addition, the relative temperature sensitivity (*S*_r_), temperature uncertainty (*δT*), and temperature resolution were also evaluated, as described in the Supporting Information. In the measurement of the temperature-dependent PL emission spectra, the possible heating effect from the used excitation light source was also excluded (Figure S28). As a result, the actual temperature measured by the core@shell nanostructures can be read by extraction from the LIR-*T* relationship. On the other hand, the photothermal conversion of plasmonic metal nanostructures can lead to temperature rise in the surrounding medium, which allows metal nanostructures to serve as heat sources in many applications, such as photothermal therapy, bioimaging, nanochemistry, and plasmonic catalysis.^67−69^ The real-time monitoring of the temperature variations in these circumstances becomes very important but often challenging. We illuminated our (Au NS)@Y(V,P)O_4_:Eu powder samples with an 808 nm laser diode at different optical power densities (Figure S29). The sample temperature rose and reached a stabilized value in a few seconds at any optical power density (Figure 6d). The temperature of the Au-free Y(V,P)O_4_:Eu sample only rose slightly upon laser illumination. With the Au NSs incorporated, sizable temperature increases were observed. In particular, the temperatures of the (Au NS4, NS5)@Y(V,P)O_4_:Eu powder samples exceeded 470 K at a power density of 5 W cm^–2^, probably because they have large absorption cross sections and their plasmon peaks are very close to 808 nm, as shown by their extinction spectra (Figure 2c). The TCL-based LIR technique of Eu^3+^ and the photothermal conversion of the Au NSs endow the core@shell nanostructures with both heating and temperature-measurement dual functionalities, which will offer many possibilities for applications in various fields.

## Conclusion

In conclusion, we have prepared (Au NS)@Y(V,P)O_4_:Eu core@shell nanostructures by a wet-chemistry approach. Highly uniform spherical core@shell nanostructures are synthesized from Au NSs of different diameters. The LSPR peaks of the core@shell nanostructures are controlled by use of Au NSs of different diameters to closely overlap with the different Eu^3+^ emission peaks, which allows the different emission peaks to be selectively enhanced. The RBRs of the five emission peaks of Eu^3+^ are determined. The statistical analysis of a total of 93 individual core@shell nanostructures indicates three key factors for the effective PL enhancement of RE ions. They are the LSPR–PL spectral overlap, the electric or magnetic nature of the dipole transitions, and the intrinsic quantum yield of the involved emission peak. As a result, the electric ^5^D_1_ → ^7^F_3_ transition (584 nm) shows the most significant PL enhancement among the five emission peaks by the electric plasmon resonance. The facile tailoring of the PL emissions of Eu^3+^ by the plasmon resonance in the core@shell nanostructures enables two applications based on the modulated LIRs, which are anticounterfeiting and optical temperature measurement. Moreover, the designed (Au NS)@Y(V,P)O_4_:Eu nanostructures also possess the dual functionalities of plasmonic photothermal conversion and optical temperature measurement. These results will offer many possibilities for potential uses in various fields.

## Methods

### Synthesis of the Au NSs

The Au NSs of different diameters were prepared by a seed-mediated growth method, as described previously.^40^ The details are described in the Supporting Information.

### Synthesis of the (Au NS)@Y(V,P)O_4_:Eu Samples

Four steps were involved in the synthesis process. The procedures for the core@shell nanostructures of different core sizes were similar. So (Au NS1)@Y(V,P)O_4_:Eu was taken as an example. The first step was the growth of Au NS1. The second step was the coating of silica on Au NS1. For silica coating, the obtained Au NS1 solution was centrifuged at a relative centrifugal force (RCF) of 4910 g for 12 min. After the supernatant was discarded, water (6 mL), CTAB solution (0.1 M, 60 μL), and NaOH solution (0.1 M, 60 μL) were added into the precipitate to redisperse the Au NSs. After the mixture solution was made uniform by hand shaking, TEOS solution (20 vol %, in ethanol, 25 μL) was injected, followed by gentle shaking for 10 s. The mixture solution was then left undisturbed overnight at room temperature. The (Au NS1)@SiO_2_ sample was thus obtained. Third, the phosphor precursor was coated on (Au NS1)@SiO_2_. The SiO_2_-coated Au NS1 solution was washed twice by centrifugation at a RCF of 3823 g for 8 min and redispersed in water (15 mL). Urea (0.15 g) was added under vigorous stirring. A total of 0.02 mmol RE(CF_3_COO)_3_, including Y(CF_3_COO)_3_ (95 mol %) and Eu(CF_3_COO)_3_ (5 mol %), was then added, followed by stirring for 10 min. The mixture solution was subsequently heated to 358 K with continuous stirring for 2 h in a three-neck round-bottom flask, which was placed in a heating jacket. When the reaction solution was cooled to room temperature, the suspension was separated by centrifugation and the precipitate was collected after being washed twice with water. The (Au NS1)@SiO_2_@Y(OH)CO_3_:Eu sample was thus produced. Fourth, a hydrothermal reaction was performed to give the Y(V,P)O_4_:Eu phosphor shell. The core@shell sample obtained in the third step was redispersed into water (20 mL) and stirred continuously. Ten mL of a solution containing NH_4_VO_3_ (0.017 mmol) and (NH_4_)HPO_4_ (0.003 mmol) was added drop by drop. The final reaction solution was immediately heated to 343 K and agitated for 10 min. It was then transferred into a Teflon bottle held in a stainless-steel autoclave and heated to 413 K for 10 h. After being cooled naturally to room temperature, the product was washed twice by centrifugation to remove the reaction medium and dried overnight at 343 K. The annealing treatment was carried out at 873 K for 2 h with a heating rate of 1 K min^–1^.

### Synthesis of the Nanoscale YPO_4_:Eu, Nanoscale and Microscale Y(V,P)O_4_:Eu Samples

The synthesis details of the nanoscale YPO4:Eu, nanoscale and microscale Y(V,P)O4:Eu Samples are described in the Supporting Information.

### Fabrication of the Anticounterfeiting Devices

A Si wafer was first cleaned with acetone and isopropanol and then spin-coated with the negative photoresist SU-8 2075. The thickness of the photoresist was ∼50 μm. The microfluidic channels were fabricated by UV photolithography (ABM Double Side Alignment System) on the coated photoresist.

### Characterization

The phase purity of the samples was determined on a Rigaku Smart Lab 9 kW powder XRD with Cu Kα radiation. Extinction spectra were measured on a Lambda 950 UV/visible/near-infrared spectrophotometer with plastic cuvettes of 1 cm optical path length. The morphology characteristics were checked with SEM (JEOL JSM7800-F) under an electron acceleration voltage of 10 kV. TEM images were taken on an FEI Tecnai Spirit microscope operating at 120 kV. HAADF-STEM imaging and elemental mapping were carried out on an FEI Tecnai G2 F20 S-TWIN FA microscope equipped with an Oxford EDX spectrometer under an electron acceleration voltage of 200 kV. The far-field scattering images were recorded on an optical dark-field microscope (Olympus, BX53M), which was integrated with a quartz-tungsten-halogen lamp (100 W) and a digital color camera (Olympus, DP73). The temperature-dependent PL spectra, excitation spectra, and decay curves were recorded on an Edinburgh FLS980 fluorescence spectrophotometer.

### Single-Particle Dark-Field Scattering and PL Spectrum Measurements

The (Au NS)@Y(V,P)O_4_:Eu nanostructure samples were dispersed in water. The resultant solutions were dropped on Si substrates at low surface number densities. After being kept on the substrate for 1 min, the drop-cast solution was blown dry with nitrogen. The measurements of the single-particle dark-field scattering spectra were carried out on a dark-field microscope (Olympus BX60) that was integrated with a quartz-tungsten-halogen lamp (100 W), a monochromator (Acton SpectraPro 2300i), and a charge-coupled device camera (Princeton Instruments Pixis 400, thermoelectrically cooled to −203 K). A dark-field objective (100×) with a numerical aperture of 0.9, corresponding to an incidence angle of 64°, was employed for both exciting the individual nanostructures and collecting the scattered light from the nanostructures. After the collected light was brought directly to the monochromator through a slit, an original spectrum was received. The scattering spectrum from an individual core@shell nanostructure was corrected by subtracting the background spectrum taken from the adjacent clean regions and then dividing it with the precalibrated response curve of the entire optical system. The single-particle PL spectra were also collected on the same microscope except that an external laser at 325 nm (Kimmon Koha IK3501R-G) was used for PL excitation. Four UV-enhanced aluminum mirrors (Thorlabs PF10-03-F01) were employed to bring the laser light to the sample, where the laser light was incident on the nanostructures at an angle of ∼60°. An achromatic quarter-wave plate (Thorlabs AQWP05M-340) was used to change the laser light from linear to circular polarization. A dark-field objective (50×) was used in the setup to collect the PL signal from the individual particles. The rest of the setup remained the same as that in the scattering measurements. A pattern-matching method was employed to correlate the same particles on both of their SEM and optical images.

### FDTD Simulations

The simulation details are provided in the Supporting Information.

## Supporting Information Available

Discussions of methods for the chemicals, Au NS synthesis, and Eu-related sample synthesis, simulations, and calculations, figures of TEM/SEM images of the core@shell and related samples, HAADF-STEM and elemental mapping images, EDX spectra, XRD patterns, and average sizes of the representative samples, scattering spectra of the (Au NS2)@Y(V,P)O_4_:Eu nanostructures and Y(V,P)O_4_:Eu NSs, simulated and measured scattering spectra of the individual (Au NS)@Y(V,P)O_4_:Eu nanostructures, temperature-dependent PL spectra and PL decay curves of the representative core@shell samples, PL spectrum of Eu^3+^ in the bulk Y(V,P)O_4_:Eu material, device fabricated with the related samples, and temperature measurements using the as-prepared samples, and tables of fitting parameters for the PL decay curves of the core@shell samples (PDF)
