# Supplementary material for: Plasmon-Controlled Selective Emission Enhancement of Eu3+ with (Au Core)@(Y(V,P)O4:Eu) Nanostructures
Source: ACS Nano. 2023 May 22;17(11):10546–59. doi: 10.1021/acsnano.3c01462 (PMC10278183; doi:10.1021/acsnano.3c01462)
Supplement: Supplementary file 1 — nn3c01462_si_001.pdf [file nn3c01462_si_001.pdf]

Supporting Information

Plasmon-Controlled Selective Emission Enhancement of Eu^{3+} with (Au Core)@($Y(V,P)O_4$:Eu) Nanostructures

Jia Zhang,[†] Xizhe Cheng,[‡] Han Zhang,[§] Jiapeng Zheng,[‡] and Jianfang Wang,[‡]*

[†]Physics Department and Jiangsu Key Laboratory of Modern Measurement Technology and Intelligence, Huaiyin Normal University, Huai'an 223300, China

[‡]Department of Physics, The Chinese University of Hong Kong, Shatin, Hong Kong SAR 999077, China

[§]School of Materials Science and Engineering, Zhejiang Sci-Tech University, Hangzhou 310018, China

*Corresponding author. Email: jfwang@phy.cuhk.edu.hk

METHODS

Chemicals. $HAuCl_4 \cdot 3H_2O$ (≥99%), $NaBH_4$ (≥98%), ascorbic acid (≥99%), and cetyltrimethylammonium chloride (CTAC, ≥98%) were purchased from Sigma-Aldrich. Cetyltrimethylammonium bromide (CTAB) was obtained from Alfa Aesar. Urea (≥99%), $Y(CF_3COO)_3$ (≥98%), $Eu(CF_3COO)_3$ (≥98%), NH_4VO_3 (≥99%), $(NH_4)_2HPO_4$ (≥99%), and tetraethyl orthosilicate (TEOS) were obtained from Aladdin (Shanghai). Deionized water with a resistivity of 18.2 MΩ cm produced by a Direct-Q5 UV water purification system was utilized in all experiments.

Synthesis of the Au NSs. The Au NSs of different diameters were prepared by a seed-mediated growth method, as described previously.[1] Briefly, a freshly prepared, ice-cold $NaBH_4$ solution (0.01 M, 0.60 mL) was injected rapidly into an aqueous solution composed of $HAuCl_4$ (0.01 M, 0.25 mL) and CTAB (0.1 M, 9.75 mL) under vigorous stirring. The resultant seed solution was kept at room temperature for 3 h. After that, the seed solution (0.12 mL) was injected into a growth solution that was made in advance by mixing CTAB (0.1 M, 9.75 mL), $HAuCl_4$ (0.01 M, 4 mL), ascorbic acid (0.1 M, 15 mL), and water (190 mL) together. The reaction solution was gently shaken for 2 min and then left undisturbed overnight at room temperature. The obtained small Au NS sample was centrifuged and redispersed into water for further use. To prepare large Au NSs, overgrowth on the obtained small Au NS sample was performed. Typically, the seed solutions of different volumes (0.12–3.55 mL) containing the small Au NSs were first mixed with CTAC solution (0.025 M, 30 mL). Ascorbic acid (0.1 M, 0.75 mL) and $HAuCl_4$ (0.01 M, 1.5 mL) solutions were then added sequentially. The mixture solution was kept in an air-bath shaker (318 K, 150 revolutions per minute) for 3 h. The resultant products were finally washed by centrifugation and then redispered into CTAB solution (0.02 M, 30 mL). $HAuCl_4$ (0.01 M, 0.2 mL) solution was subsequently injected. The obtained mixture solution was placed in the air-bath shaker (318 K, 150 revolutions per minute) for 2 h. After washing by centrifugation, the Au NS samples of different diameters were produced. Six Au NS samples were prepared. Their average diameters are 53 ± 4.4, 75 ± 4.6, 91 ± 5.5, 121 ± 7.6, 156 ± 11.5, and 194 ± 10.7 nm, respectively.

Synthesis of the Nanoscale YPO_4:Eu, Nanoscale and Microscale $Y(V,P)O_4$:Eu Samples. The precursor was first prepared. $Y(CF_3COO)_3$ (0.095 mmol) and $Eu(CF_3COO)_3$ (0.005 mmol) were dissolved into water (50 mL), followed by the addition of urea (0.5 g). The mixture solution

was heated to 358 K under continuous stirring for 2 h in a three-neck round-bottom flask, which was placed in a heating jacket. Upon cooling to room temperature, the resultant suspension was washed by centrifugation three times, and the spherical $Y(OH)CO_3$:Eu precursor particle sample was obtained. The subsequent preparation processes for the nanoscale YPO_4:Eu and $Y(V,P)O_4$:Eu samples underwent a similar procedure to that of the phosphor shell in (Au NS)@$Y(V,P)O_4$:Eu except for the different reactants, i.e., $(NH_4)_2HPO_4$ (0.1 mmol) for YPO_4:Eu, and NH_4VO_3 (0.85 mmol) as well as $(NH_4)_2HPO_4$ (0.15 mmol) for $Y(V,P)O_4$:Eu). The microscale $Y(V,P)O_4$:Eu sample was obtained from the nanoscale $Y(V,P)O_4$:Eu product by further annealing treatment at 1373 K for 2 h.

FDTD Simulations. The FDTD simulations were performed using FDTD Solutions 8.15 (Lumerical Solutions). During the simulation of the scattering spectra, an electromagnetic pulse from 400 nm to 900 nm was launched into a box containing a target nanostructure. A mesh size of 1.0 nm was used for calculating the spectra, and a mesh size of 0.5 nm was employed for calculating the charge distributions. The light source was set to be incident at an angle of 64° with respect to the normal direction of the substrate to mimic the measurement setup (numerical aperture of the objective: 0.9). The substrate used in the simulations was set to be silicon, with a refractive index of 3.6. The refractive index of the surrounding dielectric environment was set to be uniform and equal to 1.0 for air. The refractive index of the $Y(V,P)O_4$:Eu phosphor shell was set to be 1.76. The dielectric function of gold was taken from Johnson and Christy's experimental data.[2] The sizes of the (Au NS)@$Y(V,P)O_4$:Eu core@shell nanostructures were set according to the actual diameters that were measured from their SEM images.

Calculation of the S_r, δT, and Temperature Resolution values. According to the previous studies,[3,4] the relative temperature sensitivity S_r can be calculated according to the widely used equation $S_r = \frac{\Delta E}{k_B T^2}$, where ΔE is the energy difference between the two involved energy levels, k_B is the Boltzmann constant, and T is the absolute temperature. ΔE was obtained to be 1583.1 cm^{-1}. The S_r value at 300 K was then calculated to be 2.5% K^{-1} for the (Au NS4)@$Y(V,P)O_4$:Eu nanostructures. Compared with other reported optical temperature-sensing luminescent materials, such as $Ca_2LaNbO_6:Pr^{3+}$ (S_r = 0.89% K^{-1}),[5] β-$NaYF_4:Er^{3+}/Yb^{3+}/SiO_2$ (S_r = 1.64% K^{-1}),[6] $Y_2O_3:Er^{3+}/Yb^{3+}$ (S_r = 1.41% K^{-1}),[7] and $NaGd(MoO_4)_2:Tb^{3+}/Pr^{3+}$ (S_r = 2.05% K^{-1}),[8] the (Au NS4)@$Y(V,P)O_4$:Eu nanostructures show an improved sensitivity.

According to the previous studies,[9,10] the δT value can be determined according to $\delta T = |\partial T/\partial R| \cdot \delta R$, where $|\partial T/\partial R|$ can be obtained from the Boltzmann equation, and δR can be obtained by repeating the measurement of the LIR–temperature relationship. As a result, the average δT was obtained to be 1.7 K in this work.

The temperature resolution can be determined by the evaluation method that was reported in the previous work,[11] where a precision of ±1% in the determination of the fluorescence intensity was assumed. The average temperature resolution for the (Au NS4)@Y(V,P)O_4:Eu nanostructures was therefore estimated to be about ±0.8 K.

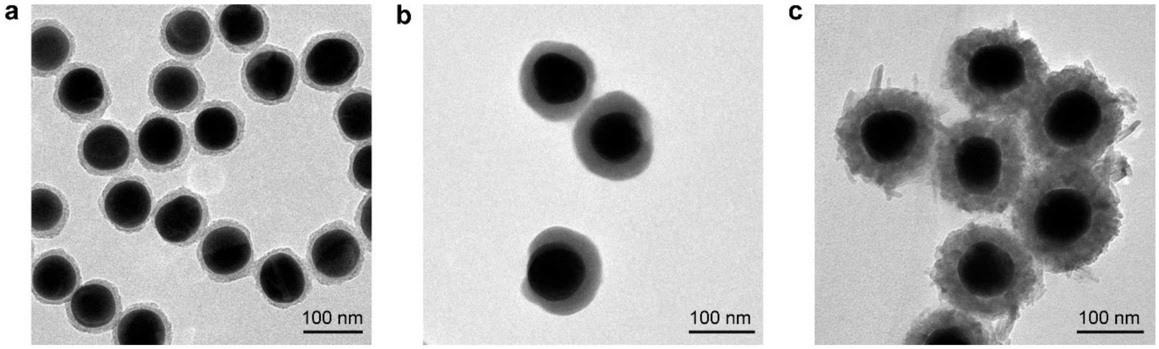

Figure S1. TEM images. (a) (Au NS2)@SiO_2. (b) (Au NS2)@SiO_2@Y(OH)CO_3:Eu. (c) (Au NS2)@Y(V,P)O_4:Eu.

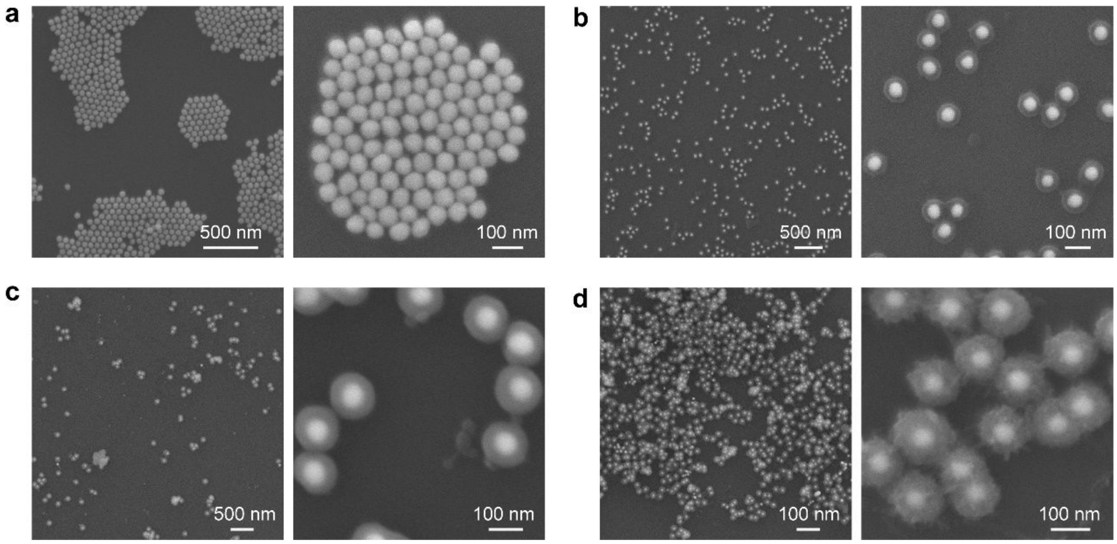

Figure S2. SEM images at two different magnifications. (a) Au NS1. (b) (Au NS1)@SiO_2. (c) (Au NS1)@SiO_2@Y(OH)CO_3:Eu. (d) (Au NS1)@Y(V,P)O_4:Eu.

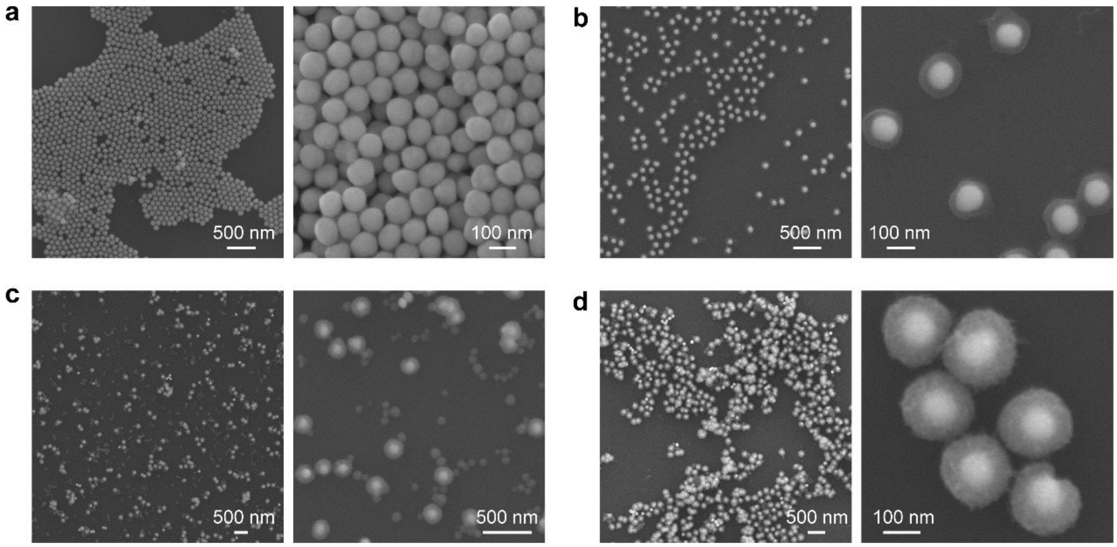

Figure S3. SEM images at two different magnifications. (a) Au NS3. (b) (Au NS3)@SiO_2. (c) (Au NS3)@SiO_2@Y(OH)CO_3:Eu. (d) (Au NS3)@Y(V,P)O_4:Eu.

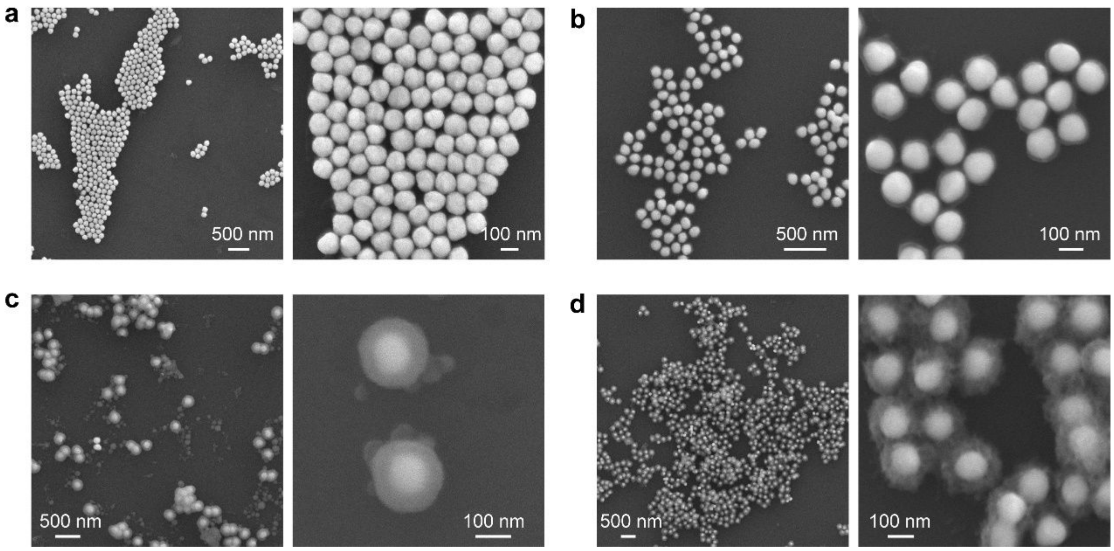

Figure S4. SEM images at two different magnifications. (a) Au NS4. (b) (Au NS4)@SiO_2. (c) (Au NS4)@SiO_2@Y(OH)CO_3:Eu. (d) (Au NS4)@Y(V,P)O_4:Eu.

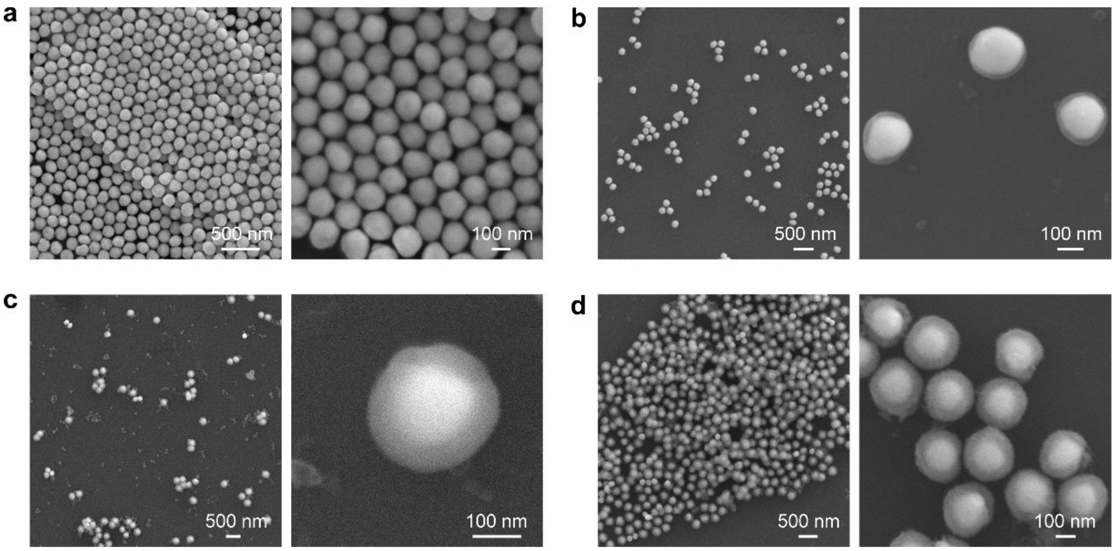

Figure S5. SEM images at two different magnifications. (a) Au NS5. (b) (Au NS5)@SiO_2. (c) (Au NS5)@SiO_2@Y(OH)CO_3:Eu. (d) (Au NS5)@Y(V,P)O_4:Eu.

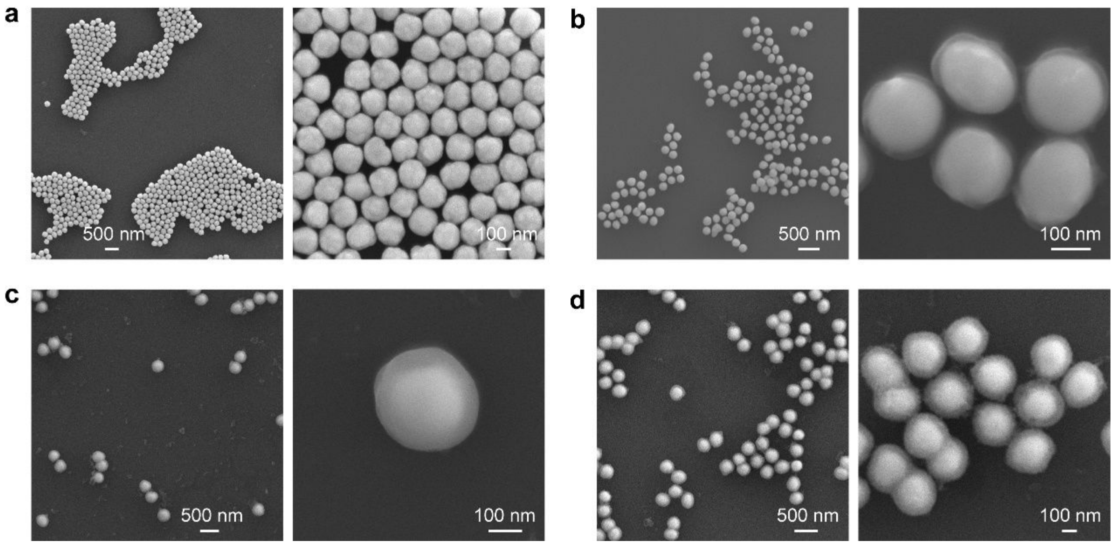

Figure S6. SEM images at two different magnifications. (a) Au NS6. (b) (Au NS6)@SiO_2. (c) (Au NS6)@SiO_2@Y(OH)CO_3:Eu. (d) (Au NS6)@Y(V,P)O_4:Eu.

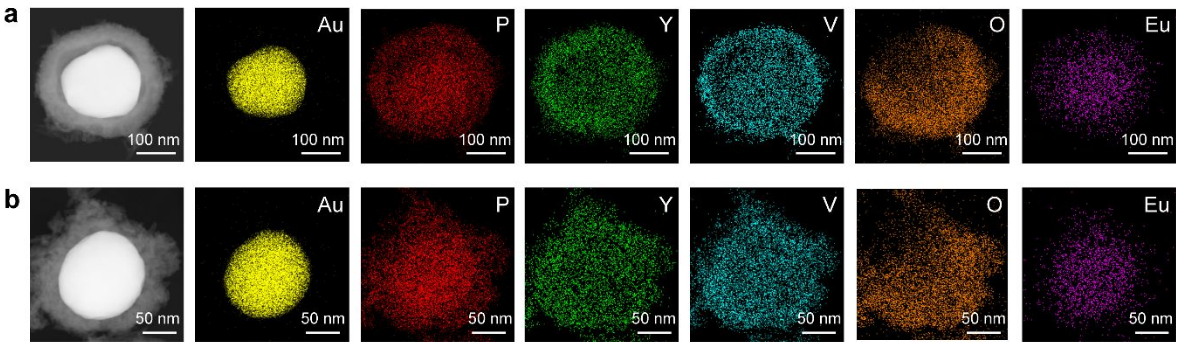

Figure S7. HAADF-STEM and elemental mapping images. (a) (Au NS6)@Y(OH)CO_3:Eu nanostructure. (b) (Au NS4)@Y(V,P)O_4:Eu nanostructure.

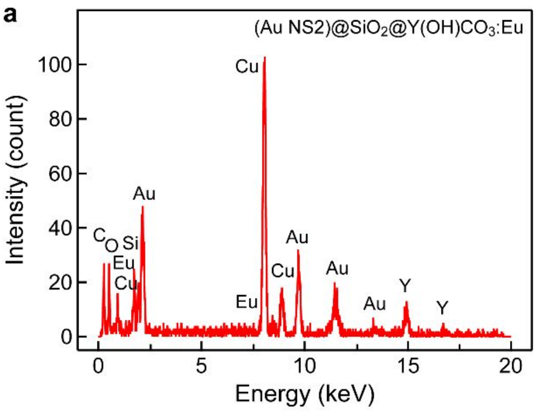

Element	Atomic proportion
Au (L)	1
Si (K)	0.511

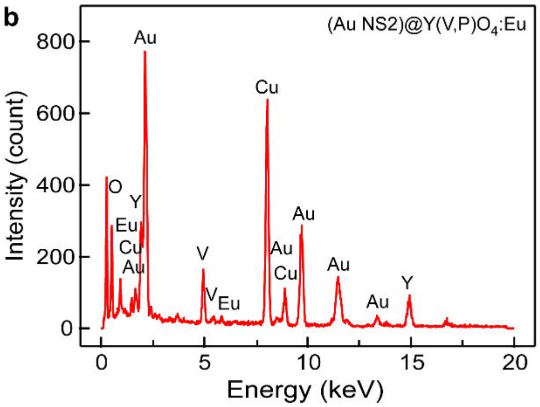

Element	Atomic proportion
Au (L)	1
Si (K)	0.043

Figure S8. EDX analysis. (a, b) EDX spectra and relative atomic ratios for (Au NS2)@SiO_2@Y(OH)CO_3:Eu (a) and (Au NS2)@Y(V,P)O_4:Eu (b). The element content of Au was set to 1.

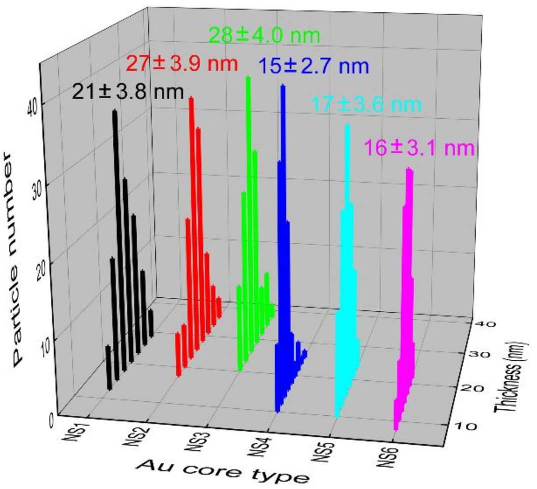

Figure S9. Thickness distributions of the SiO_2 layer in the (Au NS)@SiO_2 samples.

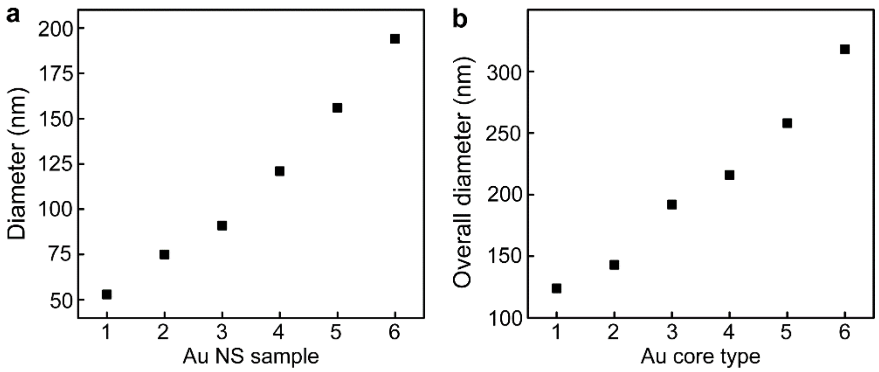

Figure S10. Particle diameter evolutions. (a) Au NS samples. (b) (Au NS)@Y(V,P)O_4:Eu samples.

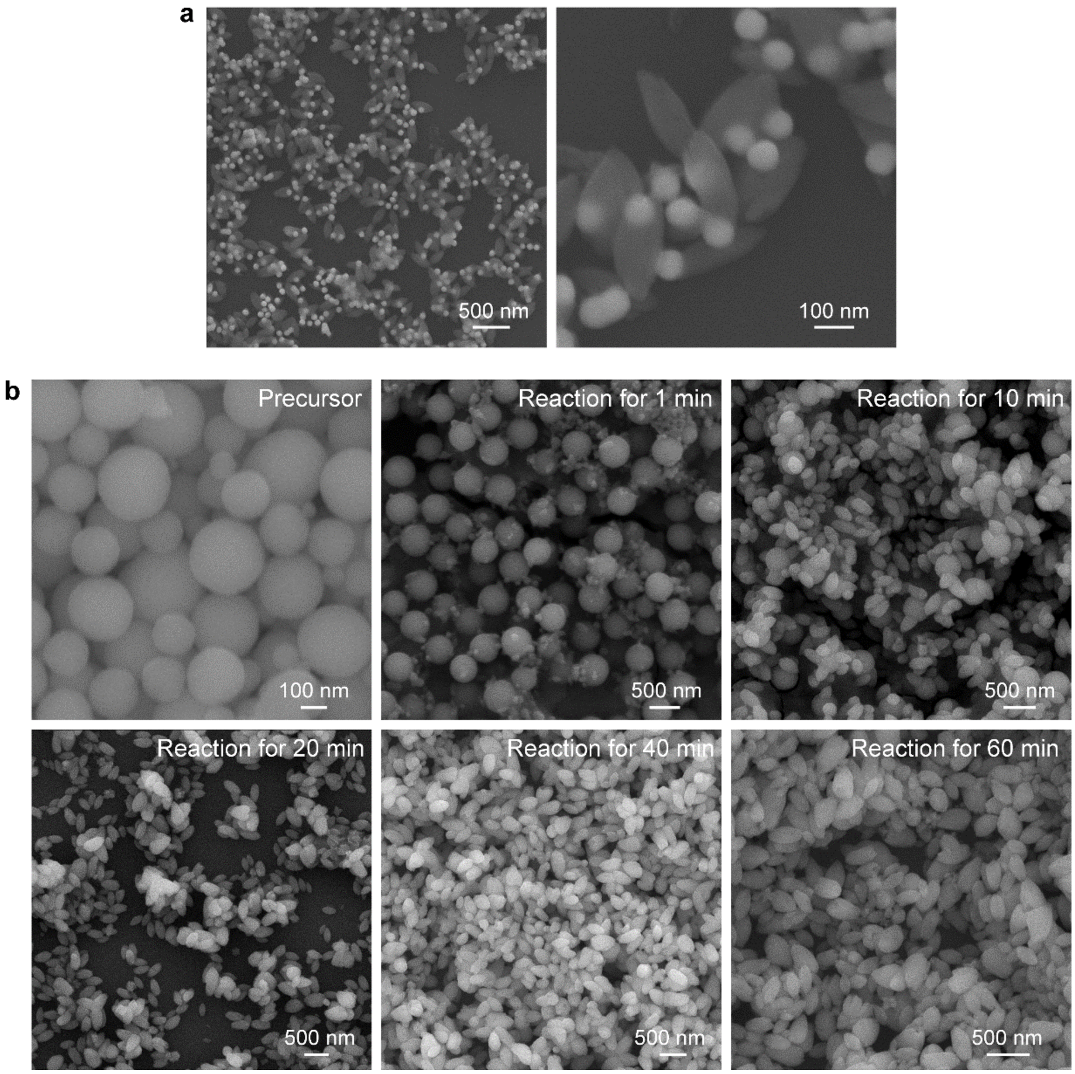

Figure S11. Morphological evolution of the phosphorus-free (Au NS)@YVO_4:Eu and YVO_4:Eu samples. (a) SEM images of (Au NS)@YVO_4:Eu. (b) SEM images for the morphological evolution of YVO_4:Eu as a function of the reaction time. The precursor is $Y(OH)CO_3$:Eu, which was prepared by a urea-based homogeneous precipitation process. The precursor was reacted with NH_4VO_3 for different periods of time.

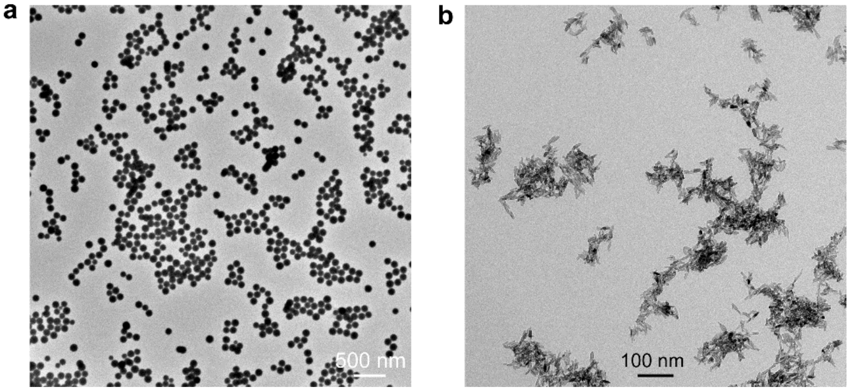

Figure S12. Morphology of the V-free YPO_4:Eu sample. (a) TEM image of the $Y(OH)CO_3$:Eu precursor. (b) TEM image of YPO_4:Eu after the hydrothermal reaction between $Y(OH)CO_3$:Eu and $(NH_4)_2HPO_4$.

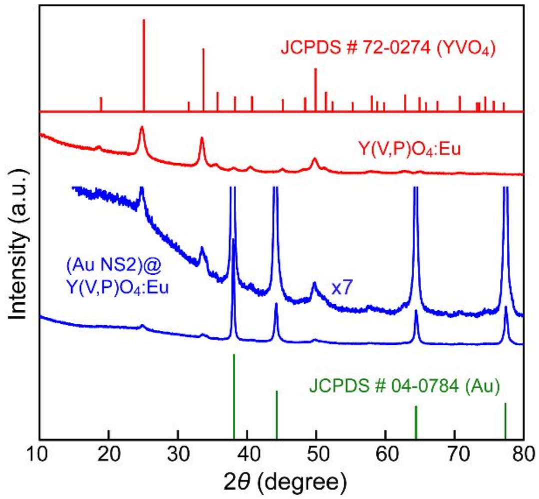

Figure S13. XRD patterns of the typical $Y(V,P)O_4$:Eu and (Au NS2)@$Y(V,P)O_4$:Eu samples. All the diffraction peaks of (Au NS2)@$Y(V,P)O_4$:Eu can be well assigned to Au (JCPDS No. 04-0784) and YVO_4:Eu (JCPDS No. 72-0274) without any impurity phase, indicating high crystallinity.

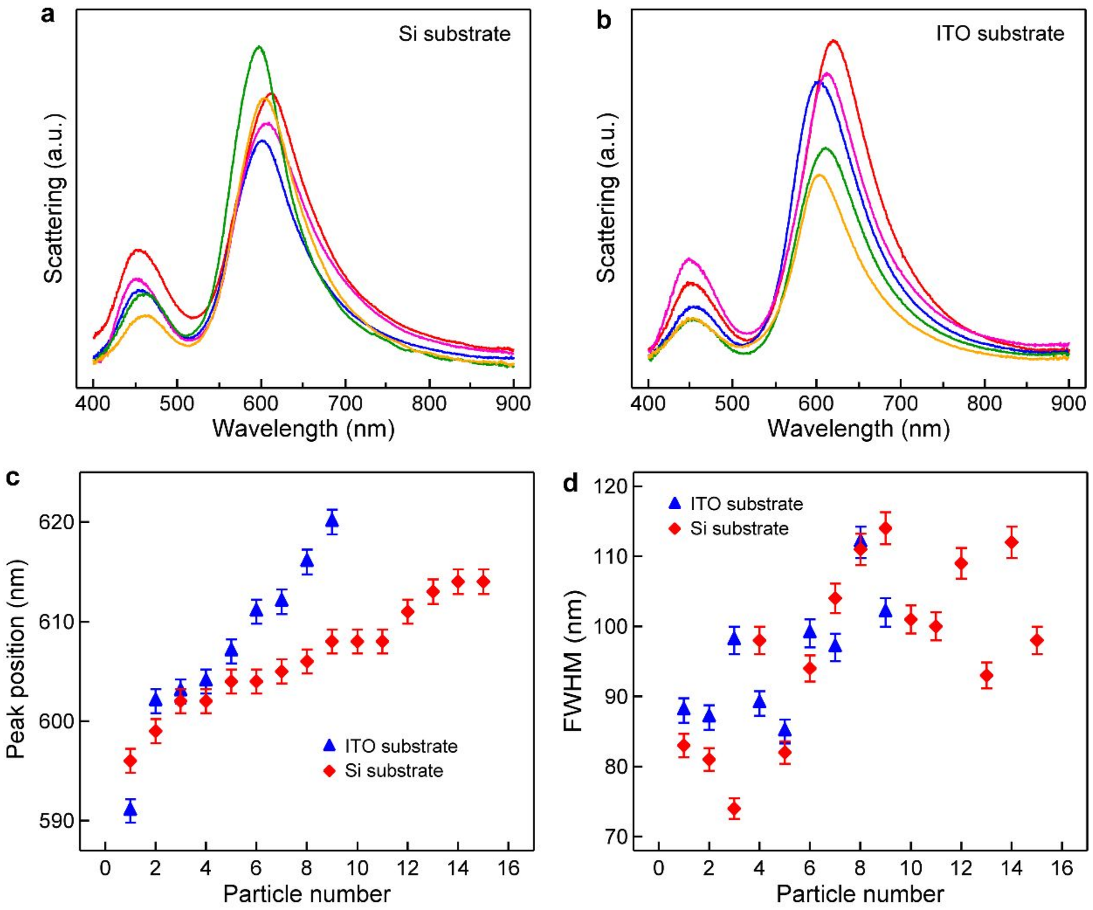

Figure S14. Scattering spectra of the (Au NS2)@Y(V,P)O_4:Eu nanostructures. (a, b) Representative scattering spectra recorded on five different (Au NS2)@Y(V,P)O_4:Eu nanostructures by use of Si (a) and ITO (b) substrates. (c, d) Distributions of the scattering peak wavelengths (c) and the full width at half maximum (FWHM) values (d) of the scattering peaks.

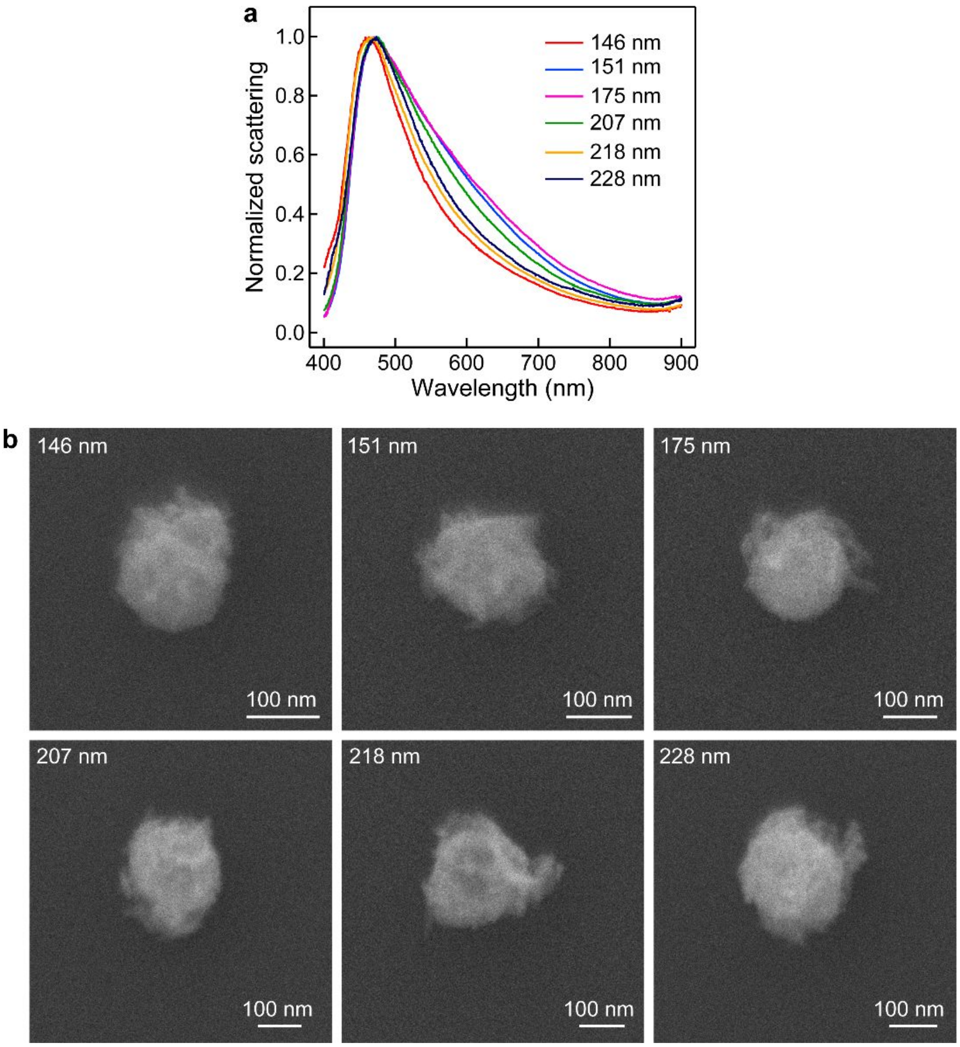

Figure S15. Scattering spectra of the individual Au-free Y(V,P)O_4:Eu NSs. (a) Representative scattering spectra taken on six individual Y(V,P)O_4:Eu NSs with different diameters. (b) SEM images of the six Y(V,P)O_4:Eu NSs, on which the scattering spectra were acquired.

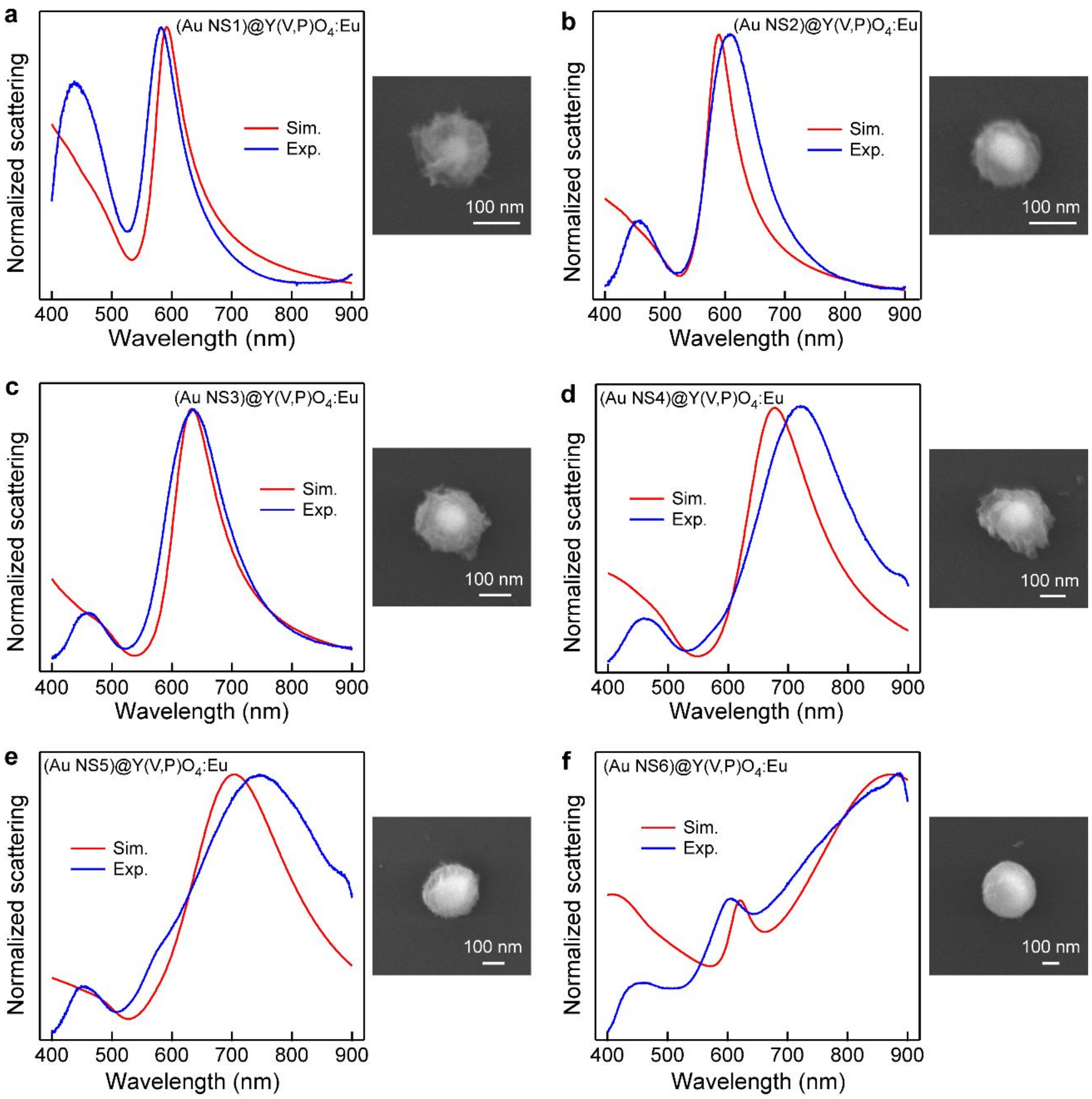

Figure S16. Simulated and measured scattering spectra of the individual (Au NS)@$Y(V,P)O_4$:Eu nanostructures and corresponding SEM images. A steep rise toward the short wavelengths below 500 nm was observed in all the simulated scattering spectra, which is different from the measured ones. The rising scattering intensities below 500 nm are attributed to the $Y(V,P)O_4$:Eu shell. In the measured scattering spectra, the weakening intensities below ~480 nm were caused by the limited detection quantum efficiencies of our optical system.

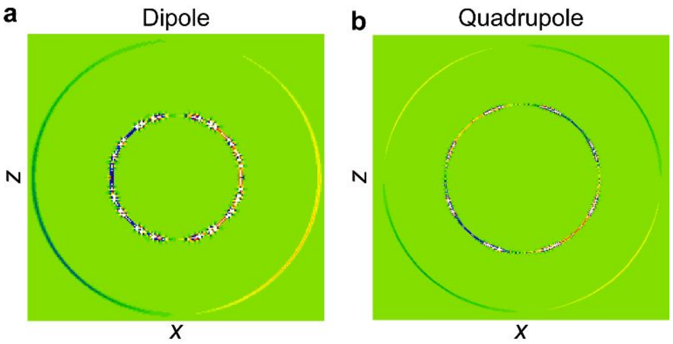

Figure S17. Plasmon resonance modes of the core@shell nanostructures. (a, b) Simulated charge distributions of the plasmon resonance peak around 650 nm for (Au NS3)@Y(V,P)O_4:Eu (a) and the shoulder plasmon resonance peak around 620 nm for (Au NS6)@Y(V,P)O_4:Eu (b). The *x*- and *z*- axes represent the spatial coordinates in the real space.

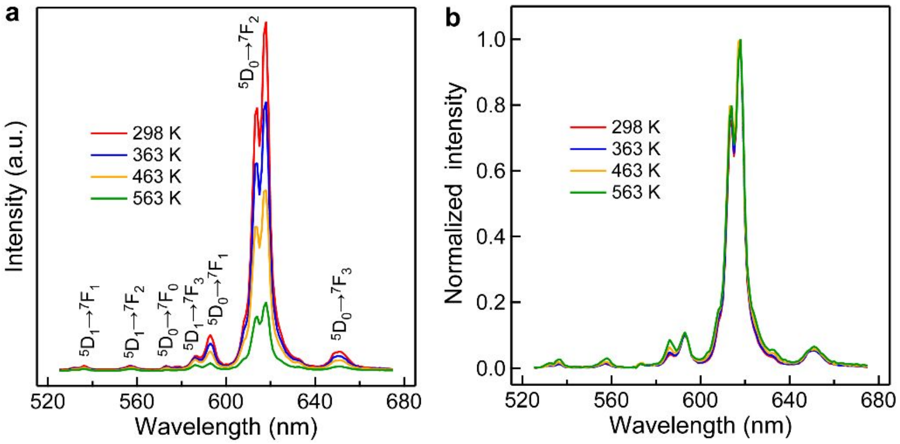

Figure S18. PL quenching. (a) PL spectra and (b) PL spectra normalized against the peak at 617 nm for (Au NS6)@Y(V,P)O_4:Eu. The spectra were measured at various temperatures. The excitation wavelength was 325 nm. The attributions of the different transitions observed in these PL emission spectra are shown in (a).

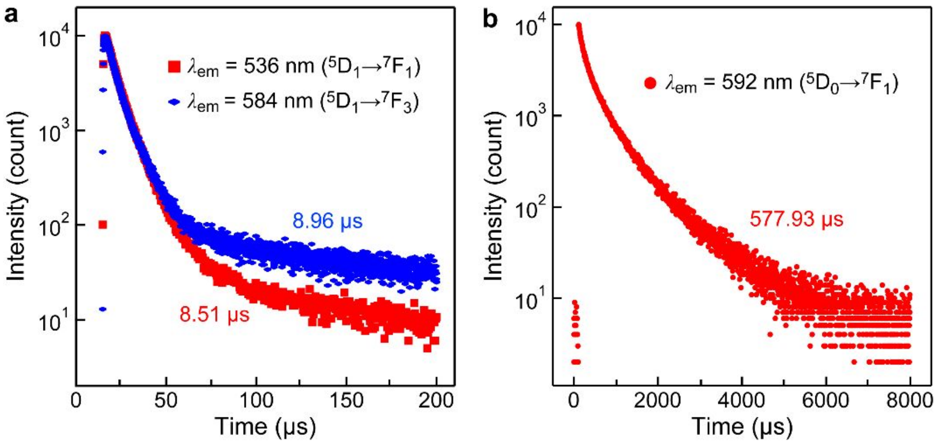

Figure S19. PL decay curves of (Au NS4)@Y(V,P)O_4:Eu. (a) Monitored at 536 nm and 584 nm. (b) Monitored at 593 nm. All the decay curves were fitted with a dual-exponential function $I = B_1\exp\left(-\frac{t}{\tau_1}\right) + B_2\exp\left(-\frac{t}{\tau_2}\right)$, where I is the emission intensity, τ_1 and τ_2 are the two decay lifetime components, B_1 and B_2 are the fitting parameters.

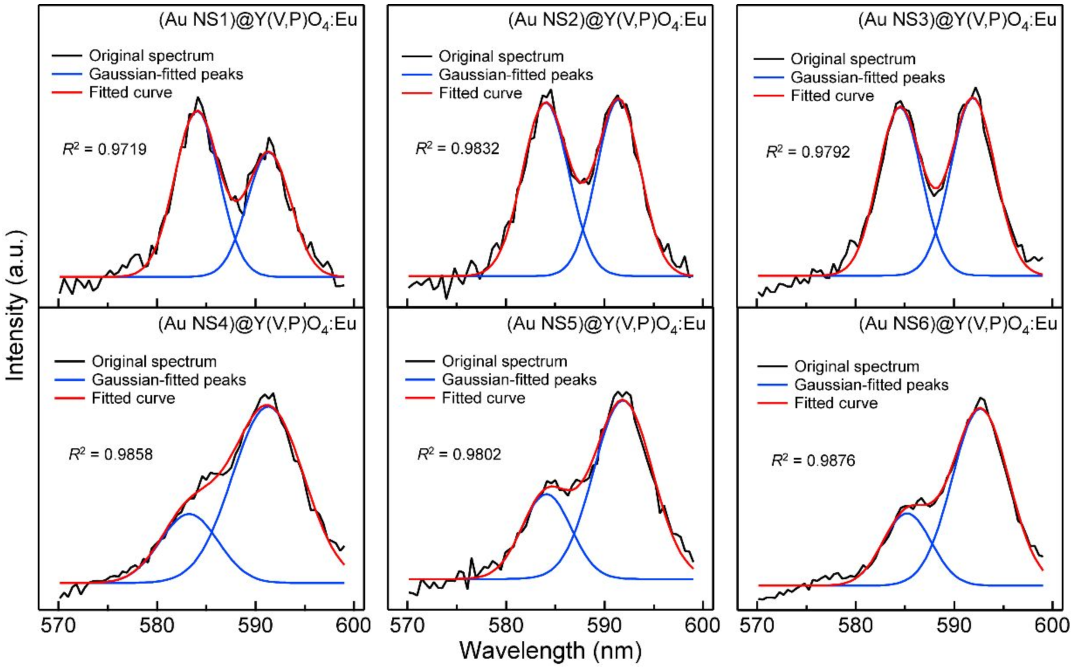

Figure S20. Gaussian fitting of the PL spectra in the range from 570 nm to 599 nm for the individual (Au NS)@Y(V,P)O_4:Eu nanostructures. The portions of the PL spectra were taken from the corresponding ones shown in Figure 3.

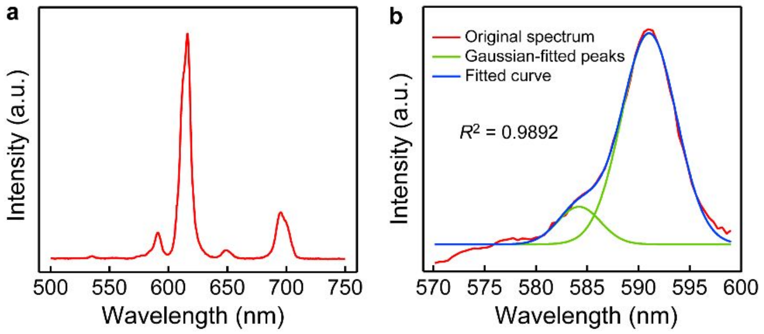

Figure S21. PL spectrum of Eu^{3+} in the bulk $Y(V,P)O_4$:Eu material. (a) Overall PL spectrum. (b) Gaussian fitting of the PL spectrum in the range from 570 nm to 599 nm. The excitation wavelength is 325 nm.

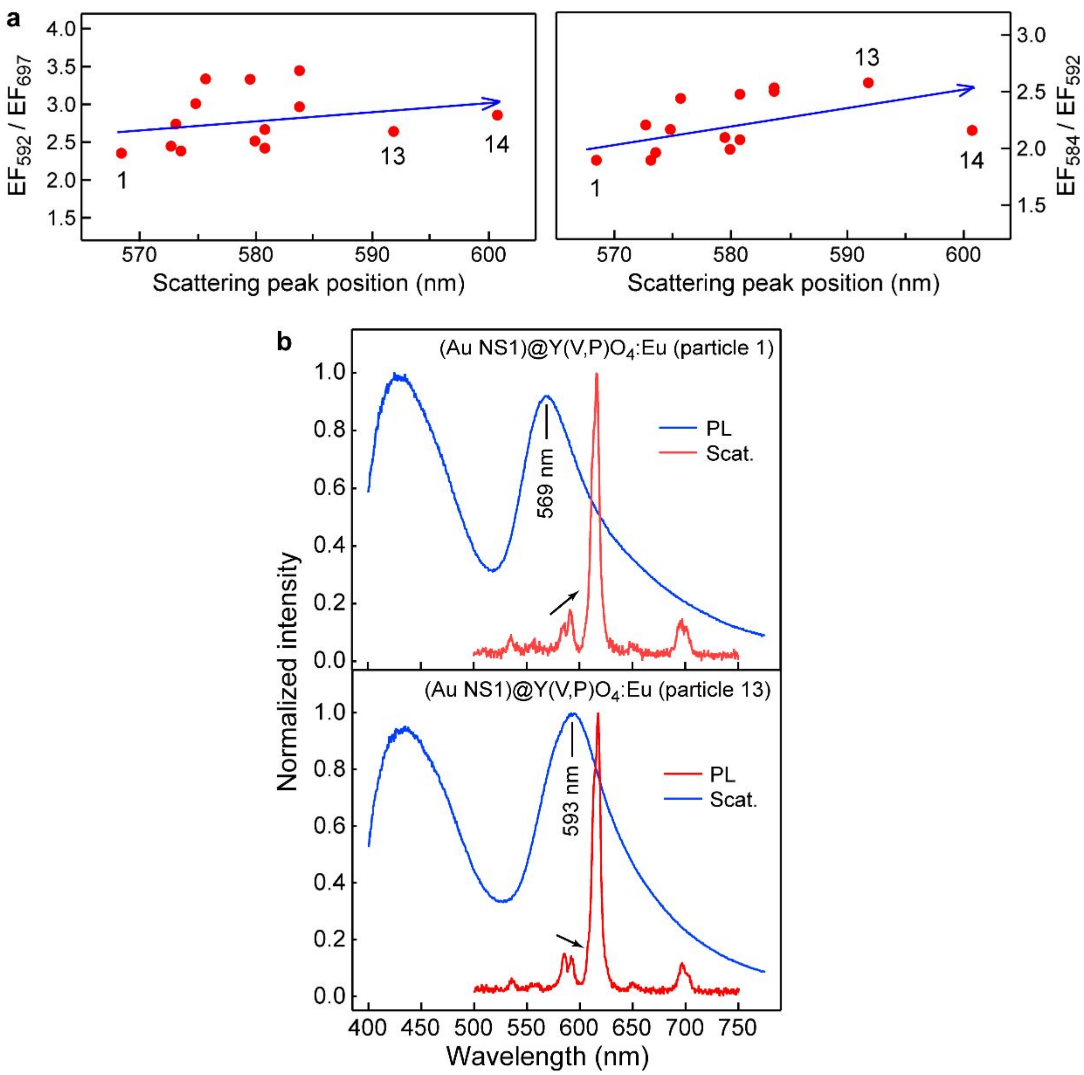

Figure S22. Comparison of the enhancement for the electric and magnetic dipole transitions of Eu^{3+} by the electric plasmon resonance. (a) Dependences of EF_{592}/EF_{697} and EF_{584}/EF_{592} of Eu^{3+} on the plasmon wavelength. The numbers 1, 13, and 14 denote the 1st, 13th, and 14th (Au NS1)@Y(V,P)O_4:Eu nanostructure. (b) Normalized PL and scattering spectra for the 1st and 13th (Au NS1)@Y(V,P)O_4:Eu nanostructure.

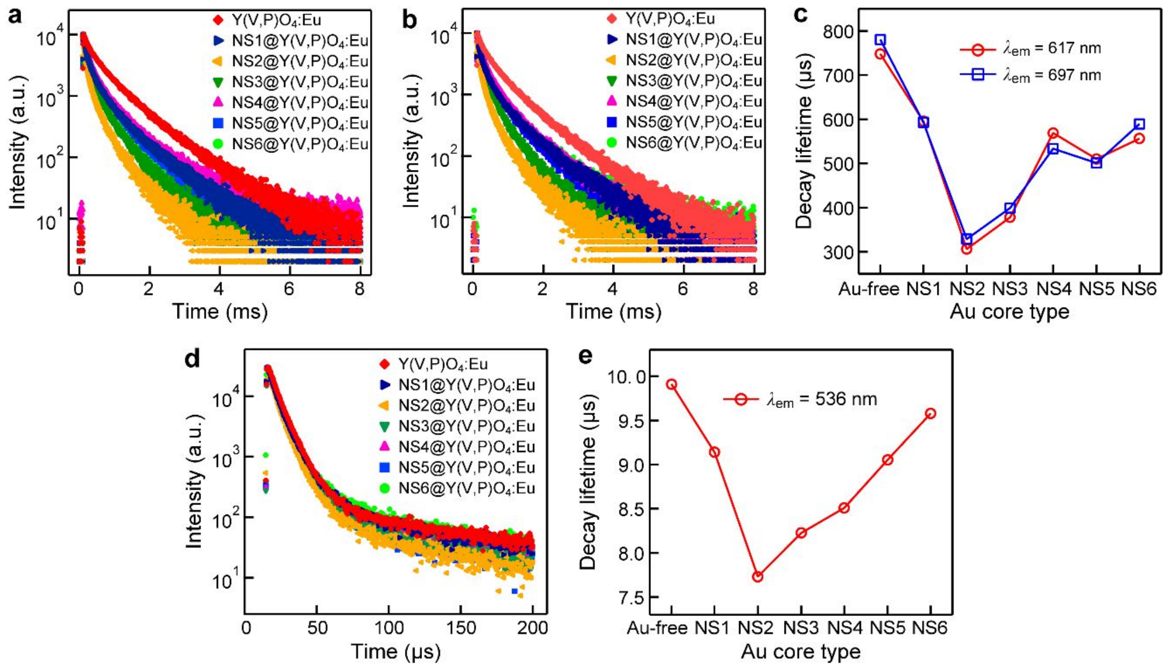

Figure S23. Effect of the plasmon resonance on the decay lifetimes of Eu^{3+}. (a, b) Decay curves of $Y(V,P)O_4$:Eu and (Au NS1–NS6)@$Y(V,P)O_4$:Eu by monitoring at 617 nm (a) and 697 nm (b). (c) Decay lifetime changes as functions of the Au core for the 617 nm and 697 nm peaks. (d) Decay curves of $Y(V,P)O_4$:Eu and (Au NS1–NS6)@$Y(V,P)O_4$:Eu by monitoring at 536 nm. (e) Decay lifetime changes as a function of the Au core for the 536 nm peak.

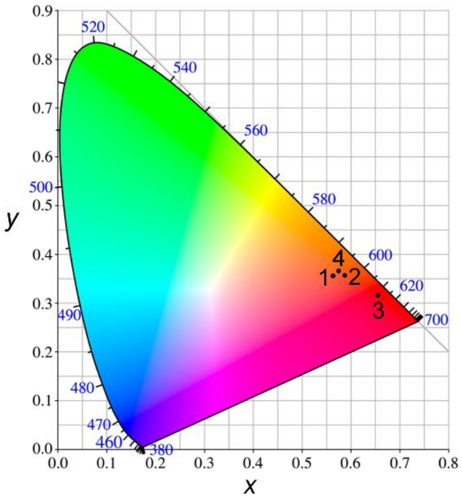

Figure S24. Difference of the colors of the four square quadrants. The colors of the four square quadrants in Figure 5b are indicated with the points 1 to 4 in the Commission International del'Eclairage (CIE) chromaticity diagram, respectively.

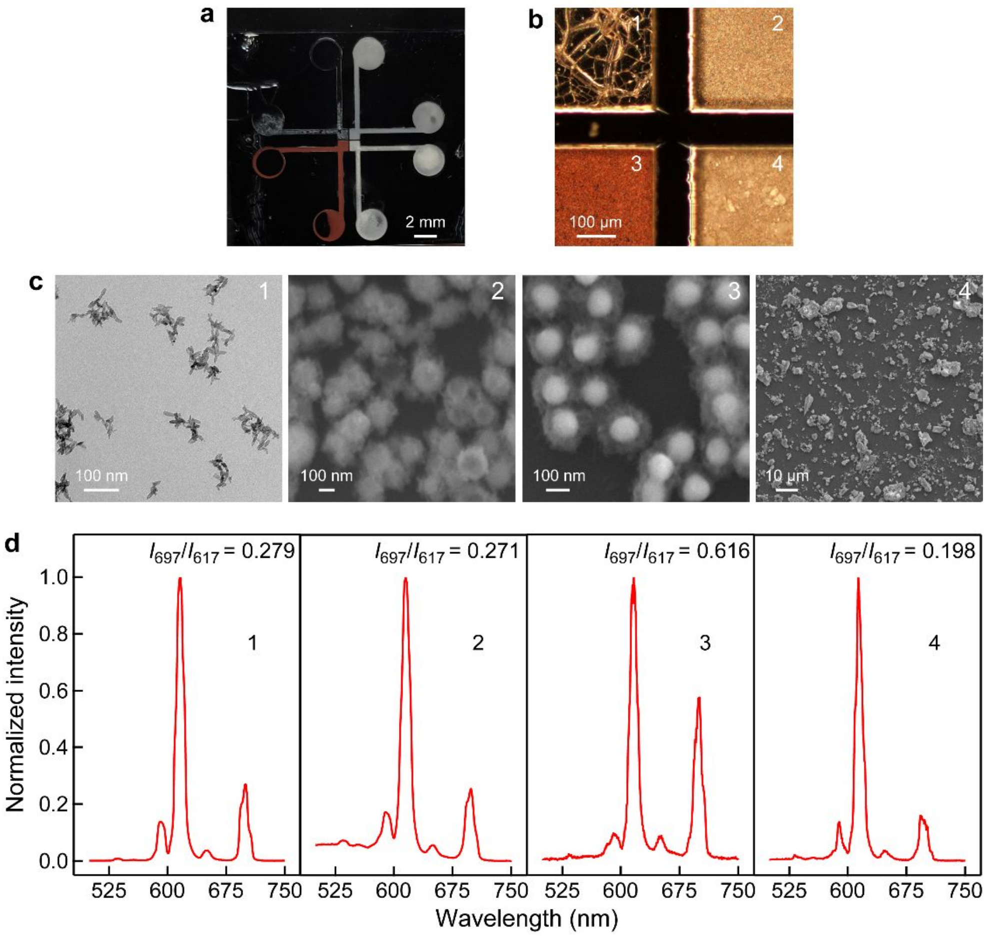

Figure S25. Device fabricated with the related samples of different morphologies. (a) Fabricated square device under daylight. The fabrication process was the same as that for the device shown in the main text. (b) Optical image of the four square quadrants at the center. The luminescent materials used to fill in the squares 1–4 are nanoscale YPO_4:Eu, nanoscale Y(V,P)O_4:Eu, nanoscale (Au NS4)@Y(V,P)O_4:Eu, and microscale Y(V,P)O_4:Eu, respectively. (c) TEM (for 1) and SEM (for 2–4) images of the samples. (d) PL spectra measured on the four square quadrants in the fabricated device. The excitation wavelength was 325 nm.

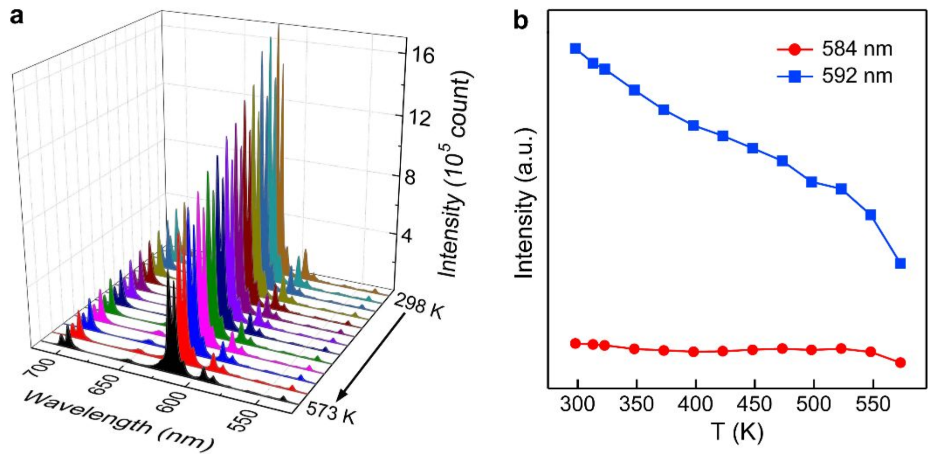

Figure S26. Temperature-dependent PL spectra. (a) PL spectra of (Au NS4)@Y(V,P)O_4:Eu measured at various temperatures. (b) Integrated intensities of the 584 nm and 592 nm emission peaks as functions of the absolute temperature. The intensities of the 584 nm and 592 nm emission peaks were obtained by integrating the emission spectra in the ranges of 580–588.5 nm and 588.5–600 nm, respectively.

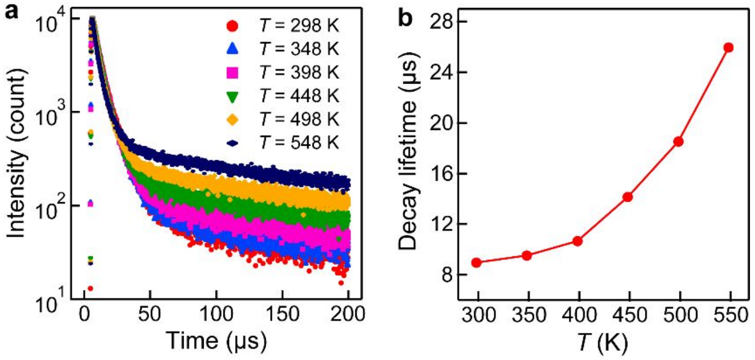

Figure S27. Dependence of the PL decay lifetime on temperature for (Au NS4)@Y(V,P)O_4:Eu. (a) Decay curves of (Au NS4)@Y(V,P)O_4:Eu at various temperatures. The monitoring wavelength was 584 nm. (b) Decay lifetime from the $^5D_1$ level of Eu^{3+} as a function of the absolute temperature.

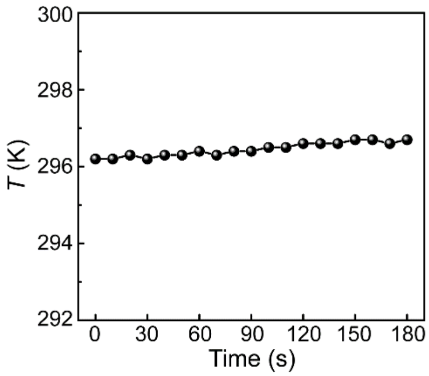

Figure S28. Dependence of the sample temperature on the irradiation time of the 325 nm excitation light source.

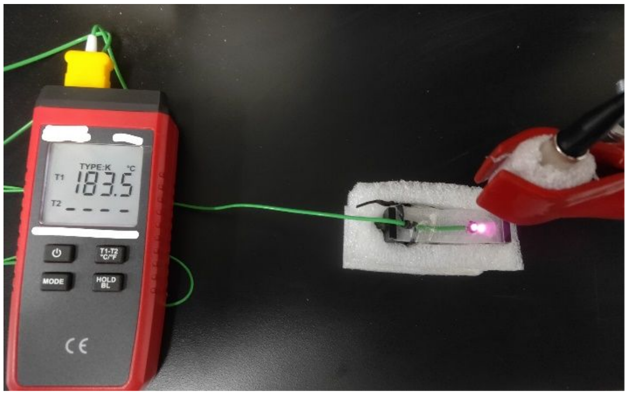

Figure S29. Temperature measurements using the (Au NS)@Y(V,P)O_4:Eu samples during plasmonic photothermal conversion. The thermocouple probe of the thermometer was buried in the powder sample. The powder sample as well as the thermocouple probe was sandwiched between two quartz substrates. After illumination for several seconds with the 808 nm laser diode at a certain optical power density, the temperature of the sample was stabilized and the final temperature was obtained from the thermometer.

Table S1. Fitting Parameters of the PL Intensity Decay Curves for (Au NS4)@$Y(V,P)O_4$:Eu

λ_{em} (nm)	τ_1 (μs)	τ_2 (μs)	B_1	B_2
536	6.35	19.62	23057.3	1449.3
584	5.89	21.21	8777.5	611.7
592	238.68	845.69	4165.9	1489.7

Table S2. Fitting Parameters of the Decay Curves for the Six Core@Shell Samples Monitored at 617 nm

Sample	τ_1 (μs)	τ_2 (μs)	B_1	B_2
$Y(V,P)O_4$:Eu	314.11	955.43	4303.5	2959.3
(Au NS1)@$Y(V,P)O_4$:Eu	190.12	868.18	4314.1	1403.3
(Au NS2)@$Y(V,P)O_4$:Eu	103.88	515.77	4664.8	903.8
(Au NS3)@$Y(V,P)O_4$:Eu	140.12	596.16	5100.0	1304.0
(Au NS4)@$Y(V,P)O_4$:Eu	216.88	846.14	4916.1	1598.1
(Au NS5)@$Y(V,P)O_4$:Eu	201.21	771.08	4817.8	1484.9
(Au NS6)@$Y(V,P)O_4$:Eu	201.57	840.32	5156.5	1539.8

Table S3. Fitting Parameters of the Decay Curves for the Six Core@Shell Samples Monitored at 697 nm

Sample	τ_1 (μs)	τ_2 (μs)	B_1	B_2
$Y(V,P)O_4$:Eu	338.17	986.86	4161.0	3061.2
(Au NS1)@$Y(V,P)O_4$:Eu	197.72	855.56	4221.7	1466.4
(Au NS2)@$Y(V,P)O_4$:Eu	116.21	531.94	3995.4	910.8
(Au NS3)@$Y(V,P)O_4$:Eu	144.72	617.81	4997.6	1362.5
(Au NS4)@$Y(V,P)O_4$:Eu	197.47	791.53	4716.9	1530.2
(Au NS5)@$Y(V,P)O_4$:Eu	190.68	739.43	4544.4	1531.7
(Au NS6)@$Y(V,P)O_4$:Eu	206.76	848.68	4818.7	1735.2

Table S4. Fitting Parameters of the Decay Curves for the Six Core@Shell Samples Monitored at 536 nm

Sample	τ_1 (μs)	τ_2 (μs)	B_1	B_2
$Y(V,P)O_4$:Eu	6.97	31.53	17878.8	537.9
(Au NS1)@$Y(V,P)O_4$:Eu	6.01	20.30	20893.2	1735.1
(Au NS2)@$Y(V,P)O_4$:Eu	5.50	19.29	20272.6	1114.6
(Au NS3)@$Y(V,P)O_4$:Eu	6.15	20.73	23054.0	1136.2
(Au NS4)@$Y(V,P)O_4$:Eu	6.35	19.62	23057.3	1449.3
(Au NS5)@$Y(V,P)O_4$:Eu	6.51	24.16	14983.1	679.5
(Au NS6)@$Y(V,P)O_4$:Eu	5.96	23.67	19346.1	1251.2

Table S5. Fitting Parameters of the Decay Curves for (Au NS4)@$Y(V,P)O_4$:Eu by Monitoring the 584 nm Emissions at Various Temperatures

T (K)	τ_1 (μs)	τ_2 (μs)	B_1	B_2
298	5.89	21.21	8777.5	611.7
348	5.93	27.53	8190.3	353.7
398	5.78	32.76	7701.3	300.3
448	5.51	43.95	7642.8	277.9
498	5.10	49.71	7660.5	338.3
548	4.68	58.52	8295.7	433.3

REFERENCES

(1) Ruan, Q. F.; Shao, L.; Shu, Y. W.; Wang, J. F.; Wu, H. K. Growth of Monodisperse Gold Nanospheres with Diameters from 20 nm to 220 nm and Their Core/Satellite Nanostructures. *Adv. Opt. Mater.* **2014**, *2,* 65–73.

(2) Johnson, P. B.; Christy, R. W. Optical Constants of the Noble Metals. *Phys. Rev. B* **1972**, *6,* 4370–4379.

(3) Shi, Y. C.; Yuan, M. J.; Li, J.; Li, F.; Cui, W. H.; Jiao, X. C.; Peng, Y. R.; Huang, Y. X.; Chen, L. Upconversion Properties and Temperature-Sensing Behaviors of Alkaline-Earth-Metal

Scandate Nanocrystals Doped with Er^{3+}/Yb^{3+} Ions in the Presence of Alkali Ions (Li^+, Na^+, and K^+). *Inorg. Chem.* **2022**, *61*, 5309–5317.

(4) Liang, Z.; Qin, F.; Zheng, Y. D.; Zhang, Z. G.; Cao, W. W. Noncontact Thermometry Based on Downconversion Luminescence from Eu^{3+} Doped $LiNbO_3$ Single Crystal. *Sensor. Actuators A Phys.* **2016**, *238*, 215–219.

(5) Zhang, A. Q.; Sun, Z.; Wang, Z. Y.; Jia, M. C.; Choi, B. C.; Fu, Z. L.; Jeong, J. H.; Park, S. H. Self-Calibrated Ratiometric Thermometers and Multi-Mode Anti-Counterfeiting Based on $Ca_2LaNbO_6:Pr^{3+}$ Optical Material. *Scripta Mater.* **2022**, *211*, 114515.

(6) Runowski, M.; Stopikowska, N.; Szeremeta, D.; Goderski, S.; Skwierczyńska, M.; Lis, S. Upconverting Lanthanide Fluoride Core@Shell Nanorods for Luminescent Thermometry in the First and Second Biological Windows: β-$NaYF_4:Yb^{3+}$–Er^{3+}@SiO_2 Temperature Sensor. *ACS Appl. Mater. Interfaces* **2019**, *11*, 13389–13396.

(7) Galvão, R.; Santos, L. F. dos; Lima, K. de O.; Gonçalves, R. R.; Menezes, L. de S. Single Er^{3+}/Yb^{3+}-Codoped Yttria Nanocrystals for Temperature Sensing: Experimental Characterization and Theoretical Modeling. *J. Phys. Chem. C* **2021**, *125*, 14807–14817.

(8) Gao, Y.; Huang, F.; Lin, H.; Zhou, J.; Xu, J.; Wang, Y. A Novel Optical Thermometry Strategy Based on Diverse Thermal Response from Two Intervalence Charge Transfer States. *Adv. Funct. Mater.* **2016**, *26*, 3139–3145.

(9) Baker, G. A.; Baker, S. N.; McCleskey, T. M. Noncontact Two-Color Luminescence Thermometry Based on Intramolecular Luminophore Cyclization within an Ionic Liquid. *Chem. Commun.* **2003**, 2932–2933.

(10) Zhang, J.; Chen, J. J.; Zhang, Y. N. Temperature-Sensing Luminescent Materials $La_{9.67}Si_6O_{26.5}:Yb^{3+}$–$Er^{3+}/Ho^{3+}$ Based on Pump-Power-Dependent Upconversion Luminescence. *Inorg. Chem. Front.* **2020**, *7*, 4892–4901.

(11) Peng, H. S.; Stich, M. I. J.; Yu, J. B.; Sun, L.-Y.; Fischer, L. H.; Wolfbeis, O. S. Luminescent Europium(III) Nanoparticles for Sensing and Imaging of Temperature in the Physiological Range. *Adv. Mater.* **2010**, *22*, 716–719.
